# Sequence–activity mapping and model-guided engineering of the phenol-responsive DmpR–Po σ^54^ promoter

**DOI:** 10.3389/fmicb.2026.1958192

**Published:** 2026-10-07

**Authors:** Yuxi Xu, Mingxuan Song, Wenglong Xing, Jianying Han, Jiahao Wu, Yi Cheng, Jiayi Liu, Linfeng Wang, Ji Zeng, Yu Zhao, Jinquan Guo, Shuting Cai

**Affiliations:** 1 School of Biomedical and Pharmaceutical Sciences, Guangdong University of Technology, Guangzhou, China; 2 School of Computer Science and Technology, Guangdong University of Technology, Guangzhou, China; 3 Guangdong Provincial Key Laboratory of Functional Soft Condensed Matter, School of Material and Energy, Guangdong University of Technology, Guangzhou, China; 4 Department of Breast and Thyroid Surgery, The Affiliated Panyu Central Hospital, Guangzhou Medical University, Guangzhou, China; 5 School of Integrated Circuits, Guangdong University of Technology, Guangzhou, China

**Keywords:** DeepSHAP, DmpR–Po, FACS-seq, in silico mutagenesis, machine learning, promoter engineering, σ^54^ promoter

## Abstract

Promoters are core cis-regulatory elements that determine gene expression levels and dynamic responses, and their quantitative engineering directly dictates the performance of synthetic biological systems such as whole-cell biosensors. However, the transcriptional output of complex inducible systems, exemplified by σ^54^-dependent promoters, relies on long-range coordination among distal bacterial enhancer-binding proteins, DNA architectural proteins, and RNA polymerase. The combined effects of multiple regulatory regions on promoter output remain poorly understood, which limits the cross-background transfer of local sequence rules and hinders the accurate prediction and rational design of promoter activity. The phenol-responsive DmpR–Po promoter serves as both a classical model for investigating σ^54^-dependent regulation and a high-value sensing element for environmental pollutant detection and high-throughput enzyme activity screening. Resolving sequence–activity relationships across its complete regulatory region therefore holds both mechanistic and practical significance. To overcome the barriers to rational engineering of complex promoters, we established a modular design–measure–model–guide–validate framework targeting the full DmpR–Po regulatory region. A library of 17,500 designed promoter sequences was constructed to systematically perturb the upstream activating sequence (UAS), the IHF2 region, combined UAS–IHF2 mutational backgrounds, and downstream regulatory architecture. Fluorescence-activated cell sorting coupled with sequencing yielded a high-quality sequence–activity map covering 11,940 variants. At the regional level, identical UAS and IHF2 haplotypes generally preserved their relative activity rankings across single-region and joint-variant backgrounds, and the main effects estimated independently for UAS and IHF2 could approximately predict the activity of combined variants. At the nucleotide level, however, the effects of individual substitutions were strongly constrained by the overall DNA architecture and full-sequence context. A deep learning model trained on the complete regulatory sequence captured these context-dependent relationships, achieved robust predictive performance on held-out sequences and a separately transformed and processed Batch 2 derived from the same preconstructed plasmid-library pool, and enabled model-guided prioritization of targeted local variants. Single-clone validation further showed that model-guided edits produced variable experimental outcomes, supporting candidate prioritization while indicating that individual engineering predictions require experimental validation. Collectively, this study characterizes the transferability and context-dependent constraints of regional sequence effects and establishes a data-driven framework for quantitative prediction and model-guided engineering of complex σ^54^-dependent promoters.

## Introduction

Promoter engineering provides a foundation for regulating gene expression and constructing genetic circuits in synthetic biology (Deal et al., 2024; Zhou et al., 2026). Unlike σ^70^-dependent promoters, whose basal activity is primarily determined by local core promoter elements, σ^54^-dependent promoters strictly require bacterial enhancer-binding proteins to hydrolyze ATP and drive the isomerization of the transcriptionally inactive σ^54^-RNA polymerase closed complex into an active open complex (Danson et al., 2019; Gao et al., 2020; Mueller et al., 2025). Distal upstream activating sequences (UASs) and DNA looping mediate enhancer-promoter communication, while architectural proteins such as integration host factor (IHF) reshape DNA geometry to facilitate long-range coordination between activators and polymerase (Rice et al., 1996; Watson et al., 2022; Tran and Le, 2024).

The phenol-responsive DmpR–Po system couples chemical sensing to σ^54^-dependent transcription. Upon recognizing phenol and related aromatic compounds, DmpR activates its AAA+ ATPase domain and drives transcription initiation from the Po promoter (Shingler and Pavel, 1995; Park et al., 2020). By converting aromatic compound recognition into measurable transcriptional outputs, DmpR–Po has been widely adopted to construct fluorescent or luminescent whole-cell biosensors, whose detection sensitivity, dynamic range, and output intensity can be further optimized via genetic circuit engineering (Leedjärv et al., 2006; Xue et al., 2014; Sun et al., 2023). For environmental monitoring, the system has been adapted for *in situ* quantification and bioavailability assessment of aromatic pollutants in complex environmental matrices. In metabolic engineering, its chemical responsiveness enables the construction of dynamic metabolic control circuits. Recent studies have extended DmpR–Po to *in vivo* sensing of phenol and p-cresol in commensal bacterial chassis, while host engineering and coupling to phenol-generating enzymatic reactions have improved sensor sensitivity, reduced intercellular crosstalk, and supported high-throughput screening and directed evolution of enzyme libraries(Kwon et al., 2020; Jin et al., 2024; Lee et al., 2025). Therefore, DmpR–Po serves as both a key model for dissecting σ^54^-dependent long-range transcriptional regulation and a valuable engineering module for aromatic pollutant detection and enzyme screening.

However, the engineering of the DmpR–Po system is both enabled and constrained by its complex regulatory architecture. Po promoter output is not dictated by an isolated core motif, but depends on the DmpR-binding UAS, the IHF-associated region, the relative positioning of these elements with respect to the σ^54^ core promoter, and the broader flanking sequence background (Sze et al., 2001; Watson et al., 2022). Previous work has demonstrated that IHF not only facilitates spatial communication between distal DmpR and RNA polymerase, but may also promote the formation and stabilization of the Po open complex by altering upstream DNA conformation. Consequently, even when local consensus sequences are preserved, alterations in inter-element spacing, helical phasing, or adjacent DNA sequences can substantially remodel promoter output (Carmona and Magasanik, 1996; Deal et al., 2024). This context dependence limits the efficacy of conventional engineering strategies based on consensus sequence replacement, local mutagenesis, or modular assembly, and makes it challenging to disentangle the effects of local protein–DNA recognition from those arising through DNA architecture and element positioning (Carr et al., 2017; Urtecho et al., 2019; Yu et al., 2021; Zhang et al., 2023). Accordingly, DmpR–Po engineering should not be limited to generating stronger promoters, but should aim to achieve predictable and graded control of induced output to meet diverse requirements for biosensor sensitivity, output dynamic range, and cellular burden.

High-throughput sequence-function mapping and machine learning models offer a promising route to characterize context-dependent promoter regulation and support rational sequence design (Kinney et al., 2010; Rohlhill et al., 2017; LaFleur et al., 2022; Zhou et al., 2025). In a landmark large-scale study, Kosuri et al. synthesized and quantitatively measured DNA, RNA, and protein outputs of 12,563 promoter–ribosome binding site combinations, demonstrating that regulatory element combinations can be quantitatively evaluated at library scale (Kosuri et al., 2013). Subsequent work using genomically integrated multiplexed reporter assays analyzed 10,898 σ^70^ promoter variants, revealing the combined contributions of core promoter elements, spacer sequences, and sequence context to expression output (Urtecho et al., 2019). Building on these advances, predictive models and deep learning-guided design frameworks have been applied to dissect sequence-context effects and engineer σ^70^ promoters with predefined transcription rates (Van Brempt et al., 2020; LaFleur et al., 2022; Zhang et al., 2023; Zhou et al., 2025). Together, these studies have advanced promoter engineering from low-throughput candidate screening toward an integrated workflow of large-scale measurement, quantitative modeling, and sequence design. More recently, ultra-high-throughput measurement combined with machine learning has extended this strategy from individual regulatory elements to large combinatorial genetic systems, enabling quantitative mapping of otherwise intractable design spaces (Rai et al., 2026).

Although recent work has enabled orthogonal rewiring and output tuning of σ^54^ promoters through changes in core promoter elements and spacer sequences (Liu et al., 2025), computational studies still largely focus on consensus characterization or promoter identification rather than large-scale sequence–activity modeling and targeted design of complete DmpR–Po regulatory regions (Barrios et al., 1999; Shujaat et al., 2023). As a result, systematic investigations into continuous activity prediction, mutational effect ranking, and targeted sequence design for DmpR–Po remain scarce.

To establish a systematic engineering framework for DmpR–Po, we characterized the DmpR–Po regulatory system in *Escherichia coli* BW25113 using a medium-copy pBR322-derived plasmid carrying the DmpR expression cassette and DmpR–Po–sfGFP reporter. In this experimental background, we constructed a modular variant library covering nearly 17,500 sequences across the full regulatory region and organized the study as an integrated design–measure–model–validate workflow (Figure 1A). The library systematically perturbs the UAS, the IHF2 region, combined UAS–IHF2 backgrounds, the relative position of IHF2, and sequences downstream of IHF2. Four-bin fluorescence-activated cell sorting coupled with sequencing (FACS–seq) was used to compute a continuous Weighted Score for each variant based on its fluorescence distribution. After quality control, 11,940 variants formed the core sequence-activity map for subsequent analyses. We first examined whether regional sequence effects of UAS and IHF2 are preserved across single-region and joint-variant backgrounds, and whether separately estimated effects of the two regions can approximately predict the activity of combined variants. We then investigated how IHF2 repositioning and downstream sequence alterations constrain the transferability of these regional effects. Since region-level effects, single-nucleotide substitutions, and pairwise nucleotide interactions cannot fully explain the activity variation across the 156-bp regulatory sequence, we developed an equal-weight MLP–Residual CNN ensemble model using only the full 156-bp regulatory sequence as input. We further combined in silico mutagenesis (ISM), model-attribution analysis, and independent single-clone validation to evaluate the model’s performance in predicting induced activity of novel sequences, inferring the direction of mutational effects, and ranking candidate sequences. Finally, we performed model-guided variant design and promoter engineering using high-activity sequences as parental templates, and validated enhanced promoter performance experimentally.

**Figure 1:**
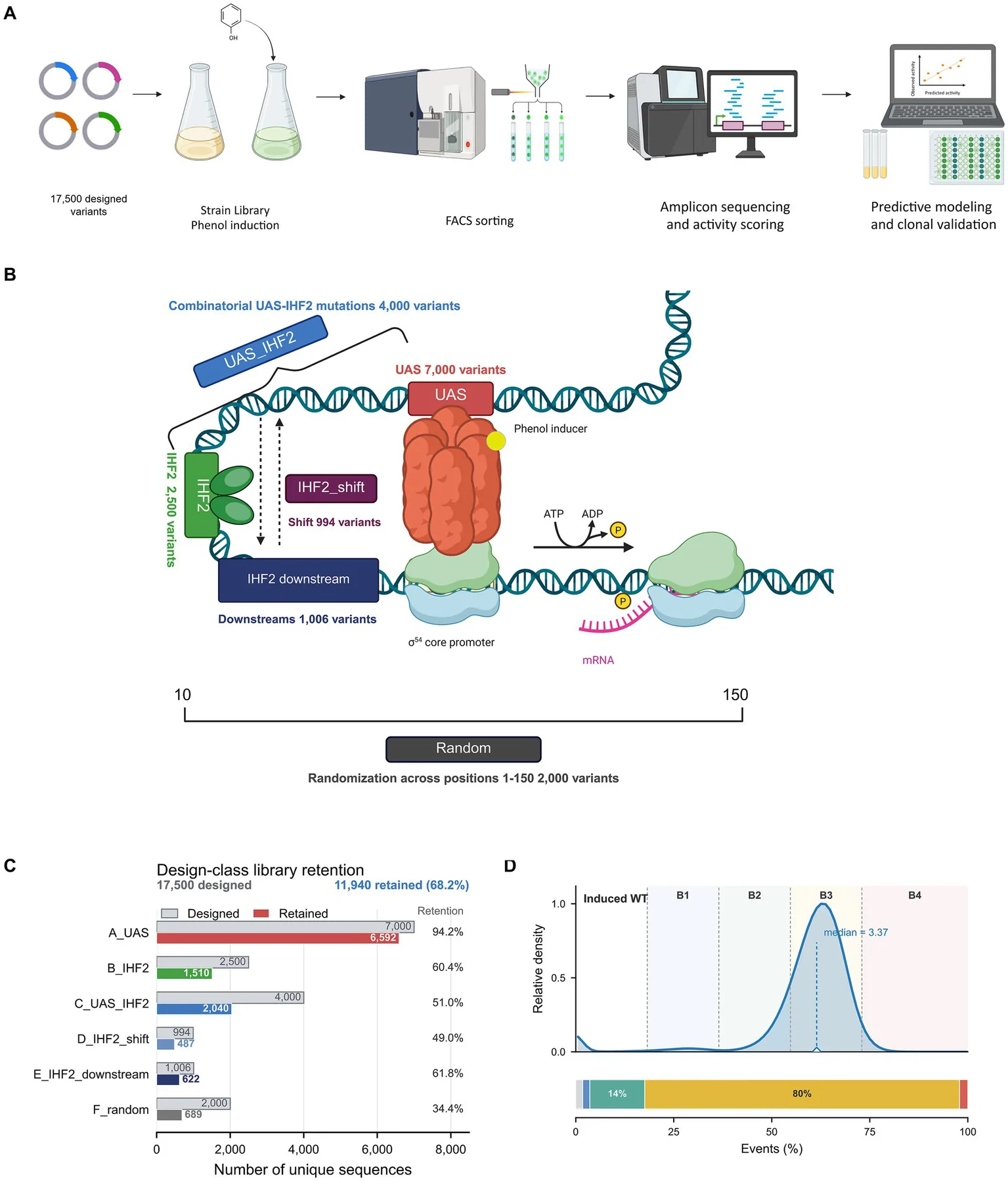
Construction and phenotypic profiling of the DmpR–Po promoter sequence-activity dataset: Overview of the DmpR–Po promoter engineering workflow and library design. **(A)** Experimental workflow for constructing and assaying promoter variants. **(B)** Regulatory architecture and modular library design of the 156-bp DmpR–Po promoter region. **(C)** Designed and retained sequence counts after sequencing, whitelist matching, and quality control. **(D)** Wild-type fluorescence distribution under the formal induced assay condition. Created in BioRender. XU, Y. (2027) https://BioRender.com/4bbn0en

## Results

### Modular design and construction of a DmpR–Po promoter variant library

All promoter-library measurements were performed in *E. coli* BW25113 using the same pBR322-derived DmpR–Po–sfGFP reporter background described above. Transcription from the DmpR-regulated Po promoter relies on long-range coordination among the upstream activating sequence (UAS), the IHF2 binding region, and the σ^54^ core promoter. To systematically dissect how local sequence composition and element positional organization collectively shape promoter activity within the intact 156-bp regulatory region, we designed six classes of variants with defined perturbation strategies (Figure 1B). Classes A_UAS and B_IHF2 introduced mutations exclusively in the UAS and IHF2 regions, respectively. Class C_UAS_IHF2 simultaneously mutated both regions while preserving their native relative positions. Class D_IHF2_shift repositioned the intact IHF2 motif via length-compensated deletion and sequence refill, maintaining the total 156-bp length of the DmpR–Po regulatory sequence to examine the composite effects associated with IHF2 repositioning and accompanying sequence reconstruction. Class E_IHF2_downstream kept IHF2 at its wild-type position but altered positions 136–150 immediately downstream of IHF2. Class F_random carried broader random perturbations across the regulatory region.

Together, these designs covered four dimensions of perturbation: local sequence changes, combined UAS–IHF2 variation, IHF2 positional shifts, and downstream sequence alterations. Detailed design coordinates, theoretical library sizes, and design rules for each class are provided in Supplementary Table 1, and the complete design whitelist is available in Supplementary Data 1. After quality filtering based on Input library read coverage and bin sequencing support, the final Batch 1 dataset comprised 11,940 unique high-quality variants (Supplementary Figure 2; Supplementary Table 2 and Supplementary Data 1), with 6,592 in A_UAS, 1,510 in B_IHF2, 2,040 in C_UAS_IHF2, 487 in D_IHF2_shift, 622 in E_IHF2_downstream, and 689 in F_random (Figure 1C).

The WT reporter showed a resolved fluorescence distribution under the formal induction condition, supporting division of the induced library into four fluorescence intervals (Figure 1D and Supplementary Figure 3). Read counts from the four FACS bins were integrated into a continuous Weighted Score (ranging from 1 to 4), where a higher score corresponds to stronger promoter activity (Supplementary Figure 2). The Weighted Score is an ordinal bin-occupancy-derived relative activity score, and the weights 1–4 represent bin order rather than calibrated fluorescence spacing. A conditional fluorescence-weighting sensitivity analysis showed highly concordant sequence ranking with the original Weighted Score (Spearman *ρ* = 0.987; Supplementary Figure 15). Input library sequencing was used only for variant coverage filtering and quality control and was not directly incorporated into the Weighted Score calculation.

The 11,940 variants covered a wide activity spectrum, with substantial activity variation both between design classes and within each class. Batch 1 thus served as the primary sequence-activity map for our core analyses. Batch 2 was generated by an independent transformation of the same preconstructed plasmid-library pool used for Batch 1, followed by separate downstream experimental processing, and was reserved to evaluate cross-batch generalizability.

Because retention differed substantially among design classes, we further examined whether sequence divergence from WT contributed to this pattern. Initial-library sequencing indicated that the apparent relationship between Hamming distance and exact-match representation was largely associated with differences among design classes, with no clear preferential recovery of lower-Hamming-distance variants within D_IHF2_shift or F_random. Across the final Batch 1 quality-control workflow, Hamming distance showed only a small additional association with retention, while the overall positional mutation profile remained broadly preserved (Supplementary Figure 16). These observations suggest that sequence divergence may contribute to retention heterogeneity but does not appear to be its predominant determinant.

### Transferability and combinatorial effects of UAS and IHF2 across sequence backgrounds

We first asked whether complete UAS and IHF2 sequence configurations retained their relative activity across different sequence backgrounds. The A_UAS, B_IHF2, and C_UAS_IHF2 classes targeted the UAS, IHF2, or both regions simultaneously, allowing direct comparison of identical regional sequences between single-region and joint-variant backgrounds (Figure 2A). We define the complete nucleotide sequence of a functional region as a regional sequence haplotype.

**Figure 2:**
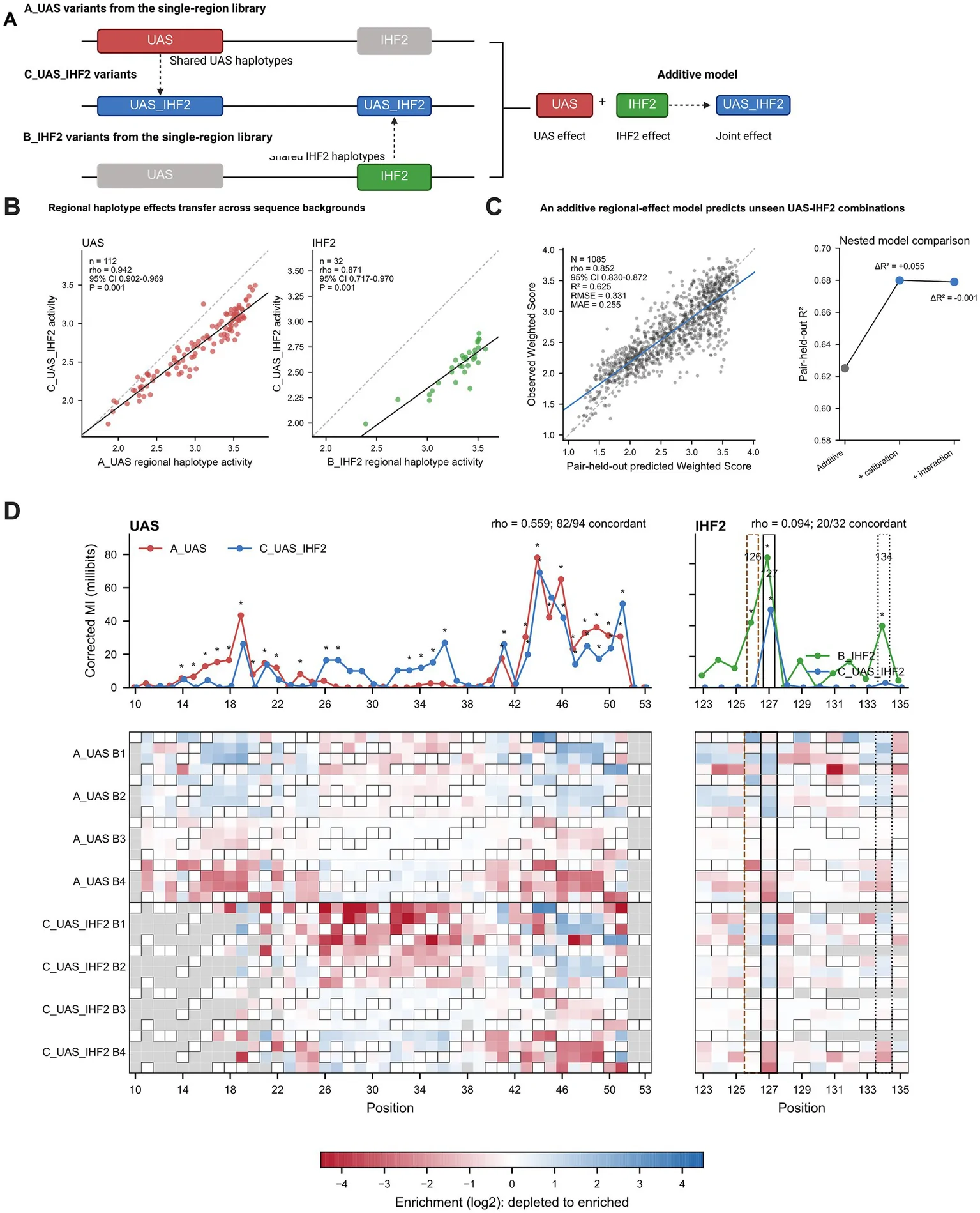
Regional UAS and IHF2 sequence effects across sequence backgrounds. Analysis of UAS and IHF2 sequence effects in single-region and joint-region backgrounds **(A)** Cross-background comparison design. **(B)** Transfer of UAS and IHF2 haplotype effects across sequence backgrounds. **(C)** Pair-held-out additive modeling of joint UAS–IHF2 variants. **(D)** Mutual-information and extreme-bin enrichment maps for nucleotide-level sequence-activity associations. These analyses summarize recoverable regional sequence effects but do not by themselves establish independent causal nucleotide effects. Created in BioRender. XU, Y. (2027) https://BioRender.com/mrh8fdq

Among 112 shared UAS haplotypes, relative activity rankings were highly preserved between the single-region and joint-variant backgrounds (Spearman *ρ* = 0.942). The 32 shared IHF2 haplotypes also showed strong rank agreement (*ρ* = 0.871), although some exhibited lower absolute activity in the joint-variant background. These relationships remained strong after accounting for sequence composition in the opposite variable region, indicating that complete UAS and IHF2 haplotypes retained substantial activity information across sequence backgrounds (Figure 2B). Detailed confidence intervals, permutation tests, and haplotype-level measurements are provided in Supplementary Figure 4 and Supplementary Data 2.

We next asked whether the effects of the two regions could be combined to predict the activity of joint UAS–IHF2 variants. Among 1,085 evaluable combinations, an additive model based on separately estimated UAS and IHF2 effects showed good agreement with measured Weighted Scores (Spearman *ρ* = 0.852, *R*^2^ = 0.625; Figure 2C). Accounting for the overall activity shift of the joint-variant background modestly improved prediction, whereas adding pair-specific interaction terms did not improve performance on held-out combinations. Thus, within the current library, the activity of most joint variants could be approximated by the contributions of the two regional haplotypes together with a global background shift, although specific epistatic interactions cannot be excluded.

Finally, we examined whether the different cross-background stability of UAS and IHF2 was distributed across each region or driven by a small number of nucleotide positions. Most comparable UAS nucleotide states (82 of 94) retained the same direction of activity effect across backgrounds, whereas this consistency was lower for IHF2 (20 of 32 states; Figure 2D). Representative positions further illustrated this difference: non-WT states at position 127 consistently reduced activity in both backgrounds, whereas position 126 showed background-dependent effects. Detailed mutual-information, enrichment, and control analyses are provided in Supplementary Figure 5 and Supplementary Data 2. Overall, complete UAS and IHF2 haplotypes generally preserved their relative activity rankings across backgrounds, but UAS showed broader nucleotide-level stability, whereas IHF2 exhibited stronger sequence-context dependence.

Overall, UAS and IHF2 haplotypes generally preserved their relative activity rankings across backgrounds, but differed substantially in position-level stability. UAS effects were distributed across multiple positions with broad directional consistency, while IHF2 had fewer stable positions and stronger context dependence.

### Effects of IHF2 positioning and downstream sequence on Po promoter activity

Having found that UAS and IHF2 effects were broadly transferable when their relative arrangement was preserved, we next asked how promoter activity changed when the IHF2 region or its downstream sequence was reconstructed. In the D_IHF2_shift design class, length-balanced deletion and sequence replacement shifted the intact 13-bp consensus-matching IHF2 element by 1–10 bp relative to its wild-type position. In the E_IHF2_downstream design class, IHF2 remained at its wild-type coordinates, whereas positions 136–150 were altered (Figure 3A). Batch 1 contained 487 D_IHF2_shift variants and 622 E_IHF2_downstream variants after quality control.

**Figure 3:**
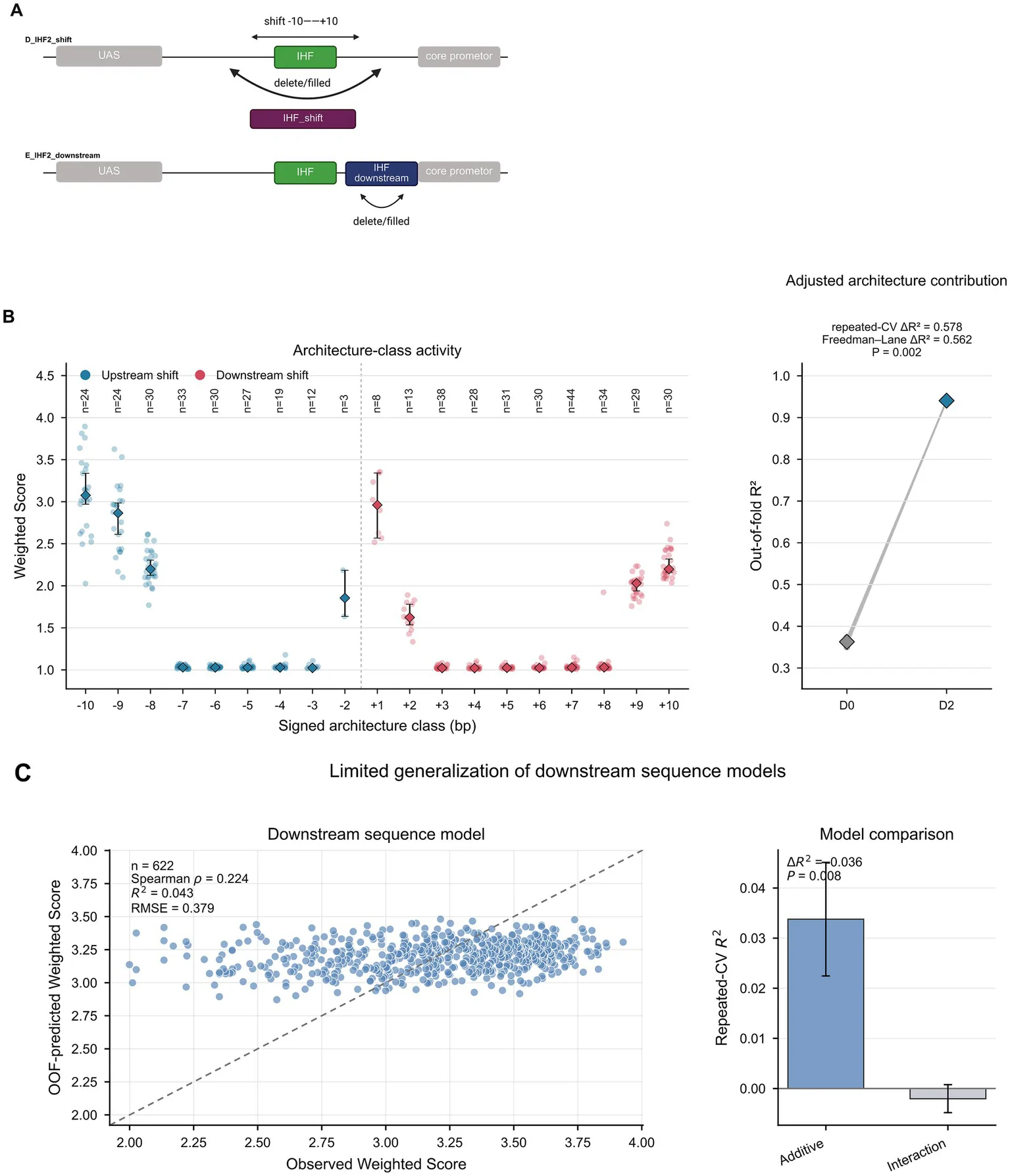
Functional consequences of IHF2-proximal architecture and downstream-sequence reconstruction. Analyses of the D_IHF2_shift and E_IHF2_downstream design classes. **(A)** IHF2 repositioning and downstream reconstruction design. **(B)** Weighted score distributions across signed IHF2-shift architecture classes and covariate-adjusted explanatory value of architecture terms. **(C)** Limited generalization of downstream sequence models and comparison of additive versus interaction models. Position-level downstream substitution effects are provided in the Supplementary Figures. The D_IHF2_shift results reflect the combined effects of IHF2 repositioning and accompanying sequence replacement rather than isolated IHF2-position effects. Created in BioRender. XU, Y. (2027) https://BioRender.com/mrh8fdq

Different D_IHF2_shift reconstruction categories occupied markedly different activity ranges (Figure 3B). The −10-bp category had the highest median Weighted Score (3.076), whereas the +4-bp category had the lowest (1.021). These category-level differences remained substantial after accounting for local sequence composition, mutation number, and Input read coverage. Detailed cross-validation and permutation statistics are provided in Supplementary Figure 6 and Supplementary Data 3. However, in the D_IHF2_shift design, the direction and distance of IHF2 movement varied together with the sequence used for length compensation. The observed category effects therefore represent the combined consequences of IHF2 repositioning and the accompanying sequence replacement and cannot be attributed independently to either the number of base pairs shifted or the direction of movement.

By contrast, sequence changes downstream of IHF2 showed weakly transferable effects across sequence backgrounds. No position–nucleotide state reached statistical significance after correction for multiple testing in the sequence-background-matched analysis. Additive and pairwise sequence models showed limited predictive performance on held-out sequences, and complementary sequence-neighbor analyses likewise indicated weak transferability across nearby sequence backgrounds (Figure 3C; Supplementary Figure 6 and Supplementary Data 3). Thus, although individual substitutions could be associated with substantial activity changes in particular backgrounds, their effects were not consistently reproduced across sequences. Overall, retaining the intact consensus-matching IHF2 element was not sufficient to preserve the same Po promoter activity. When the position of IHF2 and the accompanying adjacent sequence were altered together, different design categories occupied markedly different activity ranges. By contrast, when IHF2 remained at its wild-type coordinates, the effects of substitutions at positions 136–150 were dispersed across sequence backgrounds and could not be summarized by consistently generalizable single-nucleotide or pairwise effects.

### Full-sequence ensemble predicts promoter activity in held-out sequences and across experimental batches

Because the regional analyses left substantial activity variation unexplained, we next asked whether the complete 156-bp regulatory sequence could support accurate prediction of promoter activity. We trained sequence-only models using fixed Batch 1 training, validation, and held-out test sets (Figure 4). The MLP and Residual CNN were the strongest individual models, and their equal-weight average was selected as the final ensemble based on validation performance (Figure 5A). Neither design-class labels, sequencing coverage, Batch 2 data, nor single-clone measurements were used for model training or selection. Detailed model architectures, input representations, and training procedures are described in Materials and Methods and Supplementary Data 4.

**Figure 4:**
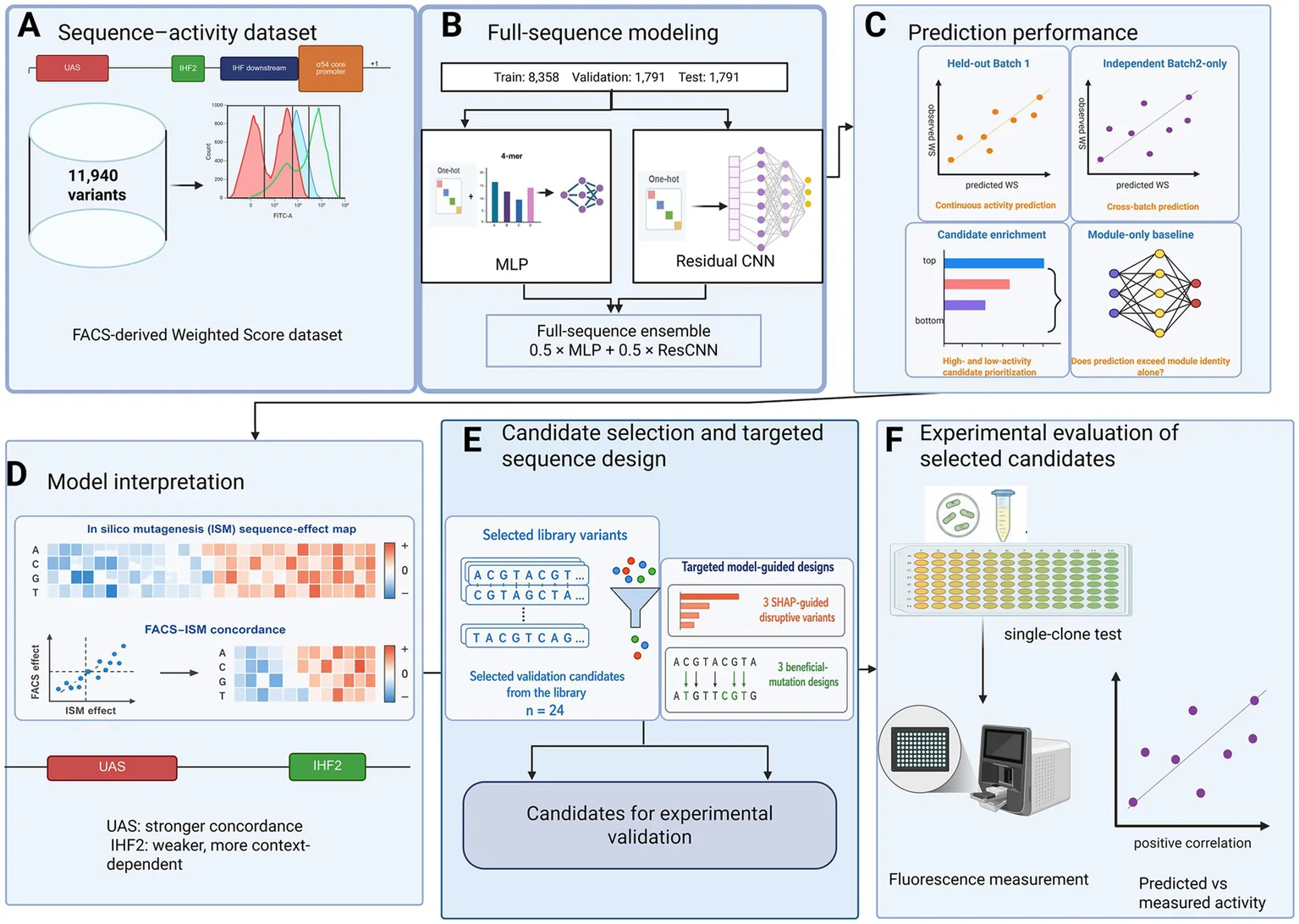
Full-sequence modeling workflow and candidate-selection framework. Overview of the sequence-only ensemble modeling and experimental-design workflow. **(A)** FACS-derived sequence-activity dataset used for model development. **(B)** Full-sequence modeling strategy using MLP and Residual CNN member models. **(C)** Prediction-performance evaluation across held-out and Batch2-only settings. **(D)** Model-interpretation analyses based on in silico mutagenesis and FACS-ISM concordance. **(E)** Candidate selection and targeted sequence-design logic. **(F)** Experimental evaluation of selected candidates. Created in BioRender. XU, Y. (2027) https://BioRender.com/mrh8fdq

**Figure 5:**
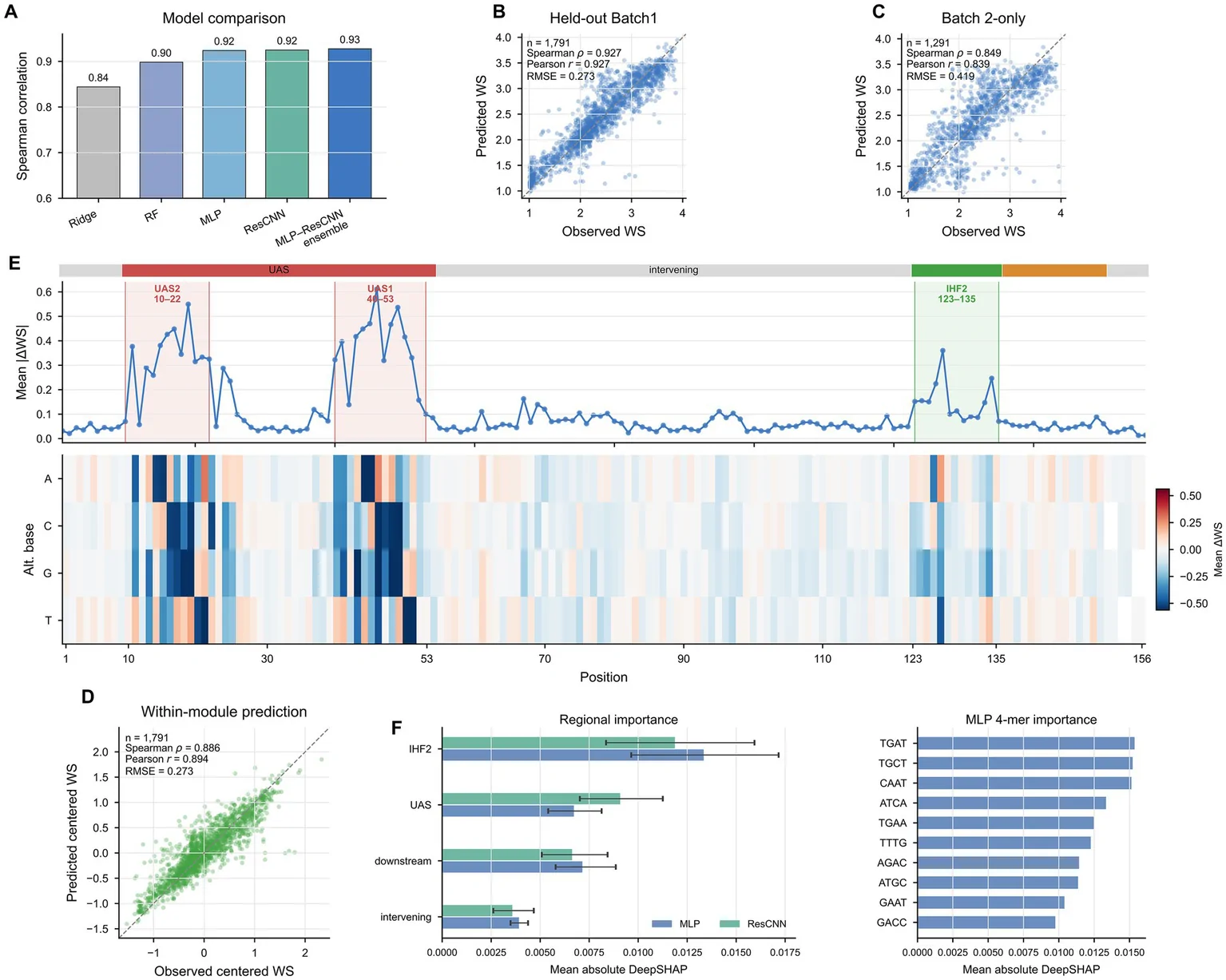
Full-sequence ensemble modeling and interpretation of DmpR–Po activity. Sequence-only modeling of FACS-derived Weighted Score using the finalized MLP–Residual CNN ensemble **(A)** Candidate model comparison showing performance of design-class-only, LightGBM, MLP, Residual CNN, and ensemble models **(B)** Held-out Batch 1 prediction accuracy for the final ensemble **(C)** Cross-batch prediction accuracy in Batch 2, including evaluation of Batch2-only sequences **(D)** Within-model prediction of centered Weighted Score **(E)** Ensemble *in silico* mutagenesis map showing position- and base-resolved predicted effects across the 156-bp regulatory sequence **(F)** Regional attribution and 4-mer importance summaries. Interpretability outputs describe model-used information and are not treated as independent mechanistic proof.

For the 1,791 held-out test sequences, the full-sequence ensemble accurately captured continuous variation in measured Weighted Scores (Spearman *ρ* = 0.928, R^2^ = 0.857; Figure 5B), including differences among sequences within the same design class. Performance also remained high under more stringent sequence-grouped holdout evaluations and exceeded nearest-neighbor regression baselines, indicating that prediction was not explained solely by highly similar training sequences (Supplementary Figure 7 and Supplementary Data 5).

To determine how much of this predictive performance could be captured by simpler sequence representations, we additionally compared the full-sequence ensemble with position-specific additive, structured pairwise, and k-mer-based baselines on the same evaluable held-out sequences. The additive model already captured substantial sequence–activity variation, and performance improved further when pairwise nucleotide-state features or short-sequence composition were included. Nevertheless, the full-sequence ensemble retained the highest overall predictive performance on the common evaluation subset (*n* = 1,721; Spearman *ρ* = 0.923, *R*^2^ = 0.845; Supplementary Table 8). These results indicate that additive, pairwise, and short-sequence-composition features account for a substantial fraction of the predictive signal, while the full-sequence model provides additional predictive resolution beyond these predefined representations.

To assess dependence on closely related training sequences, we retrained the ensemble using Hamming-1 connected-component and center-radius-1 splits. Performance remained high under both evaluations (Spearman *ρ* = 0.9211 and 0.9371, respectively) and exceeded nearest-neighbor regression baselines (Supplementary Figure 7 and Supplementary Data 5).

We next used Batch 2 in two explicitly separated ways. First, the 7,311 non-WT variants measured in both Batch 1 and Batch 2 were used to assess cross-batch reproducibility of the FACS–seq phenotype, yielding strong agreement between separately measured Weighted Scores (Spearman *ρ* = 0.8691, Pearson *r* = 0.8673, RMSE = 0.3608; Supplementary Figure 8 and Supplementary Data 6). Because only 7,311 of the 11,940 Batch 1 variants were also represented in Batch 2, we examined whether this incomplete overlap was associated with Batch 1 activity. Shared variants showed only a modest upward shift in Batch 1 activity relative to Batch1-only variants (median difference = 0.039; Cliff’s *δ* = 0.079), whereas recovery differed more substantially among design classes. Thus, Batch 2 coverage was not activity-neutral, but the overall activity-associated shift was small (Supplementary Figure 14 and Supplementary Table 9).

Additional analysis of the Batch 2-only dataset showed that the top 10% of model-ranked sequences exhibited a 5.50-fold enrichment for experimentally measured high-activity variants, whereas the bottom 10% exhibited a 7.03-fold enrichment for low-activity variants (Supplementary Figure 10 and Supplementary Data 6). Raising the minimum Input-read threshold reduced the number of retained Batch 1 variants, but the principal regional activity patterns and model-performance conclusions were not materially altered; in the shared Batch 1–Batch 2 overlap, restricting to sequences with positive bin signal in both batches gave a similar cross-batch Spearman correlation (rho = 0.8728 for n = 7,278 versus rho = 0.8691 for all 7,311 shared non-WT variants; Supplementary Figure 11 and Supplementary Data 7). Thus, despite the increased absolute prediction error across experimental batches, the model could reduce the number of candidates requiring experimental testing and prioritize both high-and low-output variants.

Because the six design classes differed in their overall activity distributions, we also asked whether the apparent predictive performance simply reflected between-class differences. A design-class-only baseline performed substantially worse than the full-sequence ensemble. After removing differences in mean activity among design classes, the ensemble still captured substantial within-class activity variation (Spearman *ρ* = 0.886, *R*^2^ = 0.796; Figure 5D and Supplementary Figure 10). Thus, the predictive performance was not explained solely by differences in average activity among the design classes. Overall, the complete 156-bp regulatory sequence supported prediction of held-out promoter activities and provided a basis for candidate prioritization. We next examined whether model-derived sequence effects agreed with experimentally measured effects in the UAS and IHF2 regions.

Overall, the complete 156-bp regulatory sequence supported continuous activity prediction for held-out DmpR–Po variants, activity ranking across experimental batches, and enrichment of candidates with extreme activity. The full-sequence ensemble can therefore be used to rank library variants and prioritize candidates for experimental validation. We next examined whether model-derived nucleotide effects and feature attributions agreed with experimentally measured effects in the UAS and IHF2 regions.

### Full-sequence models capture experimentally measured regional and nucleotide-level sequence effects

We next asked whether the sequence features learned by the full-sequence models were consistent with experimentally observed regional and nucleotide-level effects. We used in silico mutagenesis (ISM) to estimate substitution effects and DeepSHAP to examine the sequence features contributing to model predictions.

DeepSHAP analyses of both the MLP and Residual CNN highlighted the UAS- and IHF2-associated regions, and these attribution patterns were reproducible across sequence backgrounds (Figure 5F; Supplementary Figure 12 and Supplementary Data 8). Because sequence variation in the designed library was concentrated in these regulatory regions, this attribution pattern was interpreted in the context of the library design rather than as independent discovery of UAS or IHF2 regulatory architecture.

Sequence-wide ISM likewise revealed nonuniform predicted substitution effects, with prominent signals in the UAS and around IHF2 (Figure 5E). For 264 evaluated substitution states in the UAS, model-derived and experimentally estimated effects showed strong agreement (Spearman *ρ* = 0.843). Agreement was also positive for 78 IHF2 substitution states but was weaker than for the UAS (*ρ* = 0.675; Supplementary Figure 13 and Supplementary Data 8). This difference remained after accounting for the unequal numbers of evaluable substitution states between the two regions. Overall, the models captured experimentally observed sequence-effect patterns in both regions, but the agreement was stronger for the UAS. This result is consistent with the broader cross-background stability of UAS sequence effects, whereas IHF2 showed stronger sequence-context dependence.

### Independent single-clone validation of model-guided Po promoter design

We next asked whether model-based candidate ranking translated to promoter activities measured in individually constructed clones. We measured the induced activities of 24 candidate promoter sequences selected through model-guided prioritization and targeted sequence design. Paired uninduced measurements were also obtained for all 24 clones and were analyzed separately to assess clone-level basal output and induced/uninduced fold induction. Experimental activity used for comparison with model predictions was defined as the GFP/OD_600_ value under the induced condition relative to that of the wild type measured on the same plate, followed by log_2_ transformation. The corresponding uninduced activities and fold-induction values are reported in Supplementary Table 10; Supplementary Figure 17 and Supplementary Data 9 and were not used for model training or model-performance evaluation.

The prediction scores of the equal-weight MLP–Residual CNN ensemble were positively correlated with the measured induced activities of the 24 clones (Spearman *ρ* = 0.797; 95% CI, 0.494–0.942; Figure 6B). When the three severely disrupted low-activity clones DNA54_VAL_023–025 were excluded, the Spearman correlation decreased to *ρ* = 0.697 (*n* = 21); after additionally excluding DNA54_VAL_026, the correlation was *ρ* = 0.650 among the remaining 20 clones. The two measurements were also linearly correlated (Pearson *r* = 0.920, linear-fit *R*^2^ = 0.846). Because the model predicted FACS-derived Weighted Scores, whereas the single-clone phenotype was expressed as log_2_-transformed induced activity relative to the wild type, the two measurements were not on the same quantitative scale. We therefore primarily evaluated the relative ranking of the candidates. The sensitivity analysis indicates that rank agreement is stronger across the full sampled activity range and more moderate within the narrower functional range.

**Figure 6:**
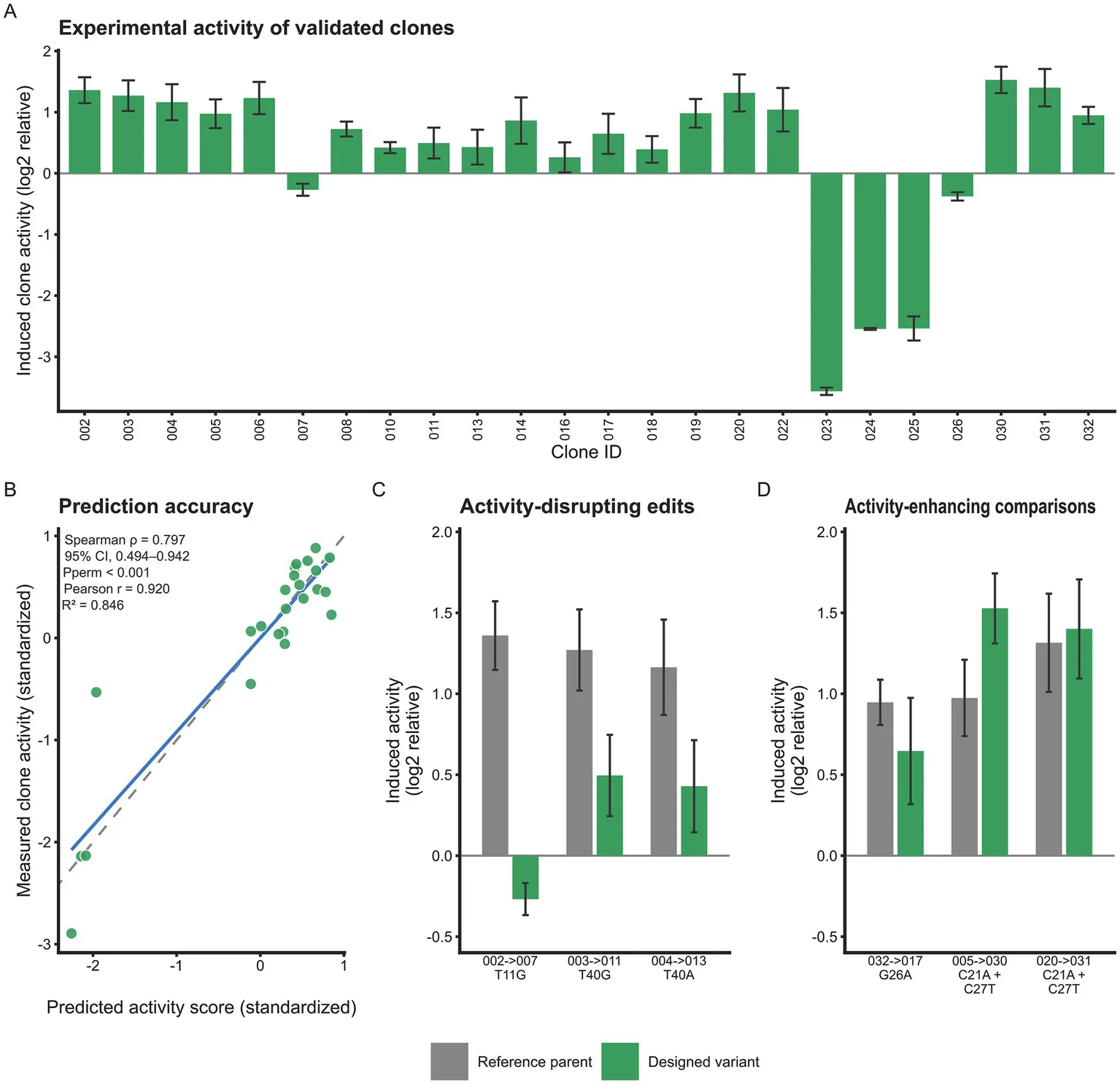
Single-clone validation of selected DmpR–Po promoter variants. Independent clone-level validation of selected promoter variants. **(A)** Induced activity of the 24 validated clones, shown as log_2_-transformed activity relative to WT. **(B)** Prediction accuracy comparing standardized ensemble-predicted activity scores with measured clone activity. **(C)** Activity-decreasing parent–variant designs, showing parental and designed-variant activities. **(D)** Activity-enhancing parent–variant designs, shown using the same clone-level phenotype and biological-replicate summary.

We designed two types of variants from high-activity parental sequences. For the first type, positions with high positive model attribution in the parental sequences were replaced with nucleotides predicted to reduce promoter activity, generating three attribution-guided activity-decreasing variants. For the second type, we prioritized local sequence edits with positive ensemble-predicted changes in Weighted Score and concordant predicted directions across the component models, generating three activity-enhancing parent–variant comparisons. These comparisons represented two distinct edit patterns: C21A + C27T in two parental backgrounds and G26A in one parental background.

The three activity-decreasing designs were DNA54_VAL_002 → 007, DNA54_VAL_003 → 011, and DNA54_VAL_004 → 013. All three variants changed in the predicted direction (Figure 6C), although the magnitude and statistical support differed among pairs. Per-pair estimates based on the biological replicates are provided in Supplementary Table 7.

Among the three activity-enhancing comparisons, DNA54_VAL_005 → 030 showed the clearest increase in induced activity (Δlog_2_ activity = 0.553, approximately 1.47-fold relative to its parent). DNA54_VAL_020 → 031 showed only a small positive change (Δlog_2_ activity = 0.085), whereas DNA54_VAL_032 → 017 changed in the opposite direction to the prediction. Per-pair statistical comparisons based on the biological replicates are provided in Supplementary Table 7. Thus, the current enhancing panel supports prioritization of individual candidate edits but does not establish a general prospective success rate for activity enhancement.

Overall, five of the six parent–variant comparisons changed in the predicted direction, but this directional agreement was treated descriptively rather than as evidence of a validated engineering procedure. The variable effect sizes and the discordant DNA54_VAL_032 → 017 result indicate that the present model is more appropriately used to prioritize candidate sequences and directional local edits than to guarantee the outcome of an individual design. Larger prospectively specified validation panels spanning multiple independent enhancing edits will be required to establish general design success rates.

## Discussion

Previous studies of the DmpR–Po system have not systematically characterized whether regional sequence effects are transferable across mutational backgrounds and how promoter architecture constrains this transferability. As a result, engineering efforts have largely relied on manipulation of individual regulatory elements, binding sites, or local sequence features, resulting in inefficient and often unpredictable design outcomes (Shingler et al., 1993; Shingler and Moore, 1994; Park et al., 2020). To address this gap, we generated a sequence-activity map of 11,940 variants spanning the complete 156-bp DmpR–Po regulatory region. Using this sequence–activity map, we evaluated the cross-background transferability and combinability of regional UAS and IHF2 effects, examined the architectural constraints imposed by IHF2-site repositioning and downstream sequence variation, and trained a full-sequence ensemble for candidate ranking and targeted directional sequence design. The full-sequence model further integrates these architecture-dependent effects and supports candidate ranking and constrained directional sequence design, providing a scalable framework for model-guided engineering of complex σ^54^-dependent promoters.

The cross-background analyses indicate that composability in the Po promoter is primarily a property of complete regional sequence configurations rather than isolated nucleotides or short motifs. When the relative positions of the UAS and IHF2 are preserved, identical regional haplotypes largely retain their activity rankings, and separately estimated effects remain informative for predicting previously unseen UAS–IHF2 combinations. Similar behavior has been observed in other bacterial regulatory systems (Otwinowski and Nemenman, 2013; Carr et al., 2017; Urtecho et al., 2019; Yu et al., 2021), where individual regulatory elements or mutational contributions account for a substantial proportion of expression variation, while departures from strict additivity persist due to sequence context and epistasis. The nucleotide-resolved enrichment patterns provide a biological basis for the stronger transferability of the UAS: the broad position-level consistency across the UAS region indicates that its transferability arises from cumulative effects across the extended activation region rather than being driven by a single critical nucleotide state. This interpretation aligns with classical work showing that DmpR recognizes an extended regulatory region encompassing UAS1 and UAS2, rather than a single independently acting short motif (Fernández et al., 1994; Sze et al., 2001).

In contrast, the IHF2 region shows a clearer distinction between transferable regional effects and context-dependent nucleotide effects. While complete IHF2 haplotypes retain cross-background activity information, individual nucleotide substitutions show far less consistency across sequence contexts. This difference is consistent with the established recognition properties of IHF, whose binding depends on both direct base contacts and indirect readout of DNA shape and deformability (Rice et al., 1996; Aeling et al., 2006; Watson et al., 2022). A complete regional haplotype preserves a combined set of sequence recognition, structural, and flanking features, whereas the functional contribution of an individual nucleotide depends on its surrounding sequence and local DNA topology. Previous work on Po has shown that IHF stimulates transcription *in vitro* and may promote or stabilize open-complex formation (Sze et al., 2001). Studies of other σ^54^-dependent promoters have further demonstrated that protein-induced DNA bending can either facilitate or impede enhancer–promoter communication, depending on bend position, element spacing, and overall regulatory geometry (Carmona and Magasanik, 1996; Valls et al., 2002; Huo et al., 2006).

These mechanistic considerations are consistent with the observed activity differences among the IHF2-region reconstruction categories: preservation of the intact 13-bp IHF2 consensus sequence alone was insufficient to maintain consistent promoter activity when the element and its neighboring sequence were jointly reconstructed. We note that in the current D_IHF2_shift design, shift direction, shift distance, and refill sequence covary, so the observed activity differences cannot be independently attributed to helical phasing, spatial displacement, or flanking-sequence replacement.

Full-sequence modeling complements regional analysis by estimating how transferable UAS and IHF2 effects are expressed within specific sequence backgrounds. Despite using different sequence representations, both the MLP and Residual CNN models assigned relatively high attribution scores to UAS- and IHF2-associated regions. Position-level attributions from both the MLP and Residual CNN were concentrated in the UAS- and IHF2-associated regions. After normalization by region length, IHF2 showed a high mean absolute attribution per position in both models, and the UAS also showed elevated attribution (Figure 5F). Because sequence variation in the designed library was concentrated in these regulatory regions, this attribution pattern was interpreted in the context of the library design rather than as independent discovery of UAS or IHF2 regulatory architecture. This correspondence should not be interpreted as evidence that the models reconstructed the molecular mechanisms of DmpR recognition, IHF binding, or open-complex formation. Attribution signals may also reflect nucleotide covariation within the designed library and other predictive sequence features.

The practical value of this framework lies in converting a large sequence–activity landscape into a tractable promoter design problem. Rather than searching the entire 156-bp sequence space without constraints, our design strategy preserves the core regulatory architecture of Po and prioritizes targeted changes within experimentally characterized sequence backgrounds. Candidate prioritization integrates full-sequence activity predictions, high-activity-associated nucleotide states identified from the experimental map, and prediction concordance between component models. The model can therefore support broad candidate ranking across the experimentally characterized activity range, supporting the design of variants tailored to different expression requirements. Compared with engineering strategies centered on single consensus motifs, this constrained full-sequence approach provides a more robust route for tuning promoter output while preserving the broader regulatory organization of Po.

For context, previous bacterial promoter-engineering studies have reported substantially larger changes after extensive promoter reconstruction; for example, Zhao et al. (2022) obtained an approximately 69-fold increase over the original Ptrc promoter. Such numerical gains are not directly comparable with the present constrained editing of DmpR–Po because promoter architecture, host background, and assay conditions differ substantially between systems.

Several limitations define the scope of the present conclusions. First, although group-aware holdout analyses supported prediction beyond direct sequence-neighbor lookup within the current library, all sequence–activity measurements were obtained in the BW25113/pBR322-derived reporter background. The quantitative effects may therefore depend on host and reporter-plasmid context, and transfer to other σ^54^-dependent promoters, hosts, plasmid backgrounds, regulatory architectures, or induction conditions remains untested. Second, the library-scale FACS–seq dataset was generated under a single induced condition and therefore characterizes induced promoter output rather than complete biosensor performance; it cannot resolve basal expression, fold induction, dynamic range, or inducer sensitivity across the full library. Third, the four-bin Weighted Score is an ordinal rather than calibrated fluorescence measure, and binning compresses within-bin fluorescence variation. Although conditional fluorescence reweighting preserved broad sequence ranking (Spearman *ρ* = 0.987; Supplementary Figure 15), fine-scale numerical differences should be interpreted cautiously. Finally, the single-clone correlation decreased from *ρ* = 0.797 for the full panel to *ρ* = 0.650 after exclusion of four severely disrupted low-activity clones, indicating more limited fine-grained ranking within the narrower functional range. Larger prospectively specified validation panels and higher-resolution phenotyping will be required to establish general design success rates.

## Materials and methods

### Bacterial strains and plasmids

Promoter-library measurements and reporter assays were performed in *Escherichia coli* BW25113. Promoter variants were cloned into a medium-copy reporter plasmid derived from pBR322, with each DmpR–Po promoter variant positioned upstream of the sfGFP reporter gene. The reporter plasmid carried an ampicillin-resistance marker, and all transformants were cultured under ampicillin selection. Unless otherwise specified, ampicillin was used at 100 μg/mL.

The DmpR expression cassette and the DmpR–Po–sfGFP reporter system were located on the same plasmid, and DmpR was expressed from its native promoter. For single-clone validation, selected promoter variants were cloned into the same pBR322-derived sfGFP reporter backbone and transformed into the same BW25113 host. Thus, pooled FACS–seq measurements and single-clone validation were performed using the same promoter–reporter configuration, plasmid-copy-number background, and antibiotic-selection conditions. The strains, reporter plasmids, and major genetic elements used in this study are listed in Supplementary Table 4.

### Phenol dose–response assay

Before FACS measurement of the promoter library, the phenol dose–response of the DmpR–Po–sfGFP reporter system was evaluated to determine the induction and sorting conditions. BW25113 cells carrying the reporter plasmid were cultured in LB medium containing 100 μg/mL ampicillin. Overnight cultures were diluted 1:100 into fresh LB medium and cultured at 30 °C and 100 rpm until the OD_600_ reached 0.2.

Phenol was then added to final concentrations of 0, 5, 10, or 20 μM, and the cultures were induced for 18 h in this preliminary dose–response assay. sfGFP fluorescence and OD_600_ were measured using a microplate reader, and fluorescence was normalized by dividing the sfGFP signal by OD_600_.

Three biological replicates were included for each condition.

Based on the dose–response curve, cell growth, and the resolution of the fluorescence distributions, 20 μM phenol was selected for the formal FACS–seq experiments (Supplementary Figure 1). The 18-h incubation was specific to the preliminary phenol-response characterization; the formal Batch 1 and Batch 2 FACS–seq experiments were performed using 20 μM phenol for 8 h.

### Library design

The promoter library comprised six design classes that perturbed the UAS region, the IHF2 region, joint UAS–IHF2 combinations, the relative position of IHF2, the sequence downstream of IHF2, or the broader full-sequence background. The six classes were designated A_UAS, B_IHF2, C_UAS_IHF2, D_IHF2_shift, E_IHF2_downstream, and F_random.

The sequence coordinates, design rules, theoretical numbers of variants, and numbers retained after quality control are provided in Supplementary Table 1. The complete design whitelist is provided in Supplementary Data 1.

### Library induction, sample preparation, and FACS sorting

The promoter library was cultured in LB medium containing 100 μg/mL ampicillin and induced with 20 μM phenol for 8 h. After induction, cultures were immediately placed on ice, and cells were collected by centrifugation at 3,000 × g for 5 min at 4 °C. The cell pellet was washed three times with ice-cold PBS, resuspended in ice-cold PBS, diluted to an OD_600_ of 0.02, passed through a 40-μm cell strainer, and maintained on ice until sorting.

FACS was performed using a BD FACSAria III cell sorter equipped with a 75-μm nozzle. The event rate was maintained below 8,000 events s^−1^. sfGFP was excited using a 488-nm laser and detected through a 525/50-nm band-pass filter. After gating the main cell population and singlets, the induced library was divided into four contiguous FITC-H fluorescence intervals, designated Bin1 to Bin4 from low to high fluorescence. The formal gating strategy and fluorescence-bin boundaries are provided in Supplementary Figure 3. Uninduced and phenol-induced cells carrying the WT DmpR–Po reporter were used as fluorescence references.

After sorting, cells collected from Bin1 to Bin4, together with the unsorted Input library, were separately inoculated into 20 mL of TB medium containing 100 μg/mL ampicillin and recovered for 10 h at 37 °C and 180 rpm. Recovered cultures were subsequently used for plasmid extraction and amplicon sequencing.

### Plasmid extraction and amplicon preparation

Plasmid DNA was extracted separately from the unsorted Input library and the four sorted fractions, Bin1 to Bin4, using a commercial plasmid-extraction kit. The region containing the complete DmpR–Po regulatory sequence was amplified using primers located in conserved sequences flanking the variable region. To reduce amplification bias, the first-round PCR was performed for 25 cycles in three parallel reactions for each sample. The parallel reactions from the same sample were pooled and purified using a PCR-purification kit.

Purified amplicons were submitted for Illumina sequencing after library preparation. No sample-specific barcode was used in the amplicon design. Primer, adapter, and index/barcode status are provided in Supplementary Table 5.

### NGS data processing

Raw paired-end FASTQ files from the Input library and the four FACS bins were processed using a workflow implemented in Galaxy. Paired-end reads were quality filtered and merged using fastp v1.3.1 (Galaxy wrapper 1.3.1 + galaxy0), with read merging enabled and unmerged reads excluded from subsequent analysis. The merged reads were processed using Cutadapt v5.2 (Galaxy wrapper 5.2 + galaxy2). In the branch used for downstream sequence counting, reads were required to contain the 5′ anchor sequence 5′-AGTATTAGAGATCAGCGTTC-3′ and the 3′ anchor sequence 5′- TCAAGCAACGGCTGTGCCAAGG-3′. Anchor matching used a maximum error rate of 0.1 and a minimum overlap of 3 bp, with indels permitted. Reads lacking either required anchor were discarded, and trimmed reads between 50 and 250 bp were retained.

Trimmed FASTQ records were converted to tabular format using FASTQ to Tabular v1.1.5 + galaxy2. Identical sequences were grouped and counted using Group v2.1.4, and sequence-count tables from the Input and four FACS-bin samples were combined using Column join v0.0.3, with absent sequence–sample combinations assigned a count of zero. The resulting tables were exported from Galaxy for exact sequence extraction, 156-bp length validation, nucleotide-content filtering, and matching against the designed promoter-library whitelist using the downstream analysis workflow.

Following export from Galaxy, fixed flanking sequences were removed and processed reads were required to yield an exact 156-bp regulatory sequence containing only A, C, G, and T. Each sequence was then matched exactly to the designed promoter-library whitelist, with no mismatches or indels permitted.

### Data filtering and weighted score calculation

Promoter variants used to construct the sequence–activity dataset were required to satisfy all of the following criteria: (i) the processed read matched exactly to a 156-bp sequence in the design whitelist and contained only A, C, G, and T; (ii) the raw Input read count was at least 5; (iii) the sum of the raw read counts across Bin1 to Bin4 was greater than 0; and (iv) the sequence could be assigned uniquely to one of the six design classes.

For variants satisfying these criteria, a pseudocount of 1 was added to the raw sequence count within each sequencing channel before channel-specific normalization to reads per million (RPM). The four bin weights were fixed as Bin1 = 1, Bin2 = 2, Bin3 = 3, and Bin4 = 4. These weights represent the ordinal identities of the four fluorescence bins rather than the physical fluorescence spacing between them. The fluorescence-weighting sensitivity analysis using the same bin-specific RPM abundances is described in Supplementary Methods 3. The Weighted Score was calculated using the pseudocount-adjusted, RPM-normalized abundances in the four sorted bins as specified below.

$$\mathrm{WS}=\frac{p({\mathrm{Bin}}_{1})+2\cdot p({\mathrm{Bin}}_{2})+3\cdot p({\mathrm{Bin}}_{3})+4\cdot p({\mathrm{Bin}}_{4})}{p({\mathrm{Bin}}_{1})+p({\mathrm{Bin}}_{2})+p({\mathrm{Bin}}_{3})+p({\mathrm{Bin}}_{4})}$$

Where p_Bin1 to p_Bin4 are the pseudocount-adjusted, RPM-normalized abundances of that sequence in the four sorted bins.

Variants with a total raw read count of 0 across all four FACS bins were assigned a missing Weighted Score and were not rescued by pseudocount addition. Sequences that failed whitelist matching, contained non-ACGT characters, had Input read count below 5, had zero total bin reads, or could not be uniquely assigned to a design class were excluded from the primary dataset. After quality control, 11,940 unique promoter variants with valid Weighted Scores were retained for analyses of design-class activity distributions and sequence-activity relationships.

The numbers and retention fractions of sequences at each processing stage are provided in Supplementary Figure 2 and Supplementary Table 2. The theoretical, detected, and retained numbers of sequences in each design class are listed in Supplementary Table 1, and the complete quality-controlled Batch 1 sequence—activity dataset is provided in Supplementary Data 1. To assess the robustness of the activity phenotype to sequence-coverage filtering, the filtering and Weighted Score calculation workflow was repeated using stricter Input and total-bin read-count thresholds. Sequence retention, agreement with the primary Weighted Scores, design-class activity distributions, and downstream model performance were compared across QC definitions. The primary analysis used the prespecified Input threshold of five reads, whereas alternative thresholds were treated as sensitivity analyses. Complete QC-sensitivity outputs are provided in Supplementary Data 7.

To assess whether sequence divergence from WT contributed to variation in library retention, Hamming distance was compared with initial exact-match representation and final retention while accounting for design class, with additional within-class analyses for D_IHF2_shift and F_random. Detailed procedures and results are provided in Supplementary Methods SM5 and Supplementary Figure 16.

### Cross-background analysis of shared regional sequence haplotypes

To test whether sequence–activity relationships within complete functional regions were retained across design backgrounds, we analyzed UAS sequences shared between A_UAS and C_UAS_IHF2 and IHF2 sequences shared between B_IHF2 and C_UAS_IHF2.

A regional sequence haplotype was defined as the complete nucleotide sequence within the corresponding coordinate interval, rather than an individual substitution state or the number of substitutions relative to WT. UAS and IHF2 coordinates used 1-based numbering within the 156-bp model-input sequence.

For each regional haplotype in a given region, complete promoter variants containing the same regional sequence were aggregated within each design class, and their mean Weighted Score was calculated.

Only haplotypes detected in both design classes and meeting the prespecified sequence-support and sequencing-coverage thresholds were retained. The final thresholds were defined by the analysis script and are reported in Supplementary Methods.

Cross-background stability of UAS haplotypes was evaluated by comparing their mean Weighted Scores between A_UAS and C_UAS_IHF2. IHF2 haplotypes were compared between B_IHF2 and C_UAS_IHF2. Rank agreement between the two backgrounds was evaluated using Spearman correlation.

Confidence intervals were estimated by sequence-level bootstrap resampling. Within each bootstrap iteration, complete 156-bp sequence records were sampled with replacement independently within the two design classes, followed by recalculation of haplotype mean Weighted Scores and the cross-background Spearman correlation. A total of 1,000 bootstrap iterations were performed for both UAS and IHF2, and the 2.5th and 97.5th percentiles were used as the 95% confidence interval.

A permutation test was used to evaluate whether the observed positive association could arise from the shared-haplotype set and design-class activity distributions. During each permutation, haplotype mean activities in the first class were held fixed, whereas the correspondence between haplotypes and mean Weighted Scores in the second class was randomized before recalculating the Spearman correlation.

This analysis evaluated whether relative activity rankings of complete regional sequences were preserved across design backgrounds.

### Additive and interaction models for UAS–IHF2 combinations

We next tested whether separately estimated UAS and IHF2 effects could be combined approximately to predict joint variants in C_UAS_IHF2. For a joint sequence containing UAS haplotype u and IHF2 haplotype v, the additive prediction was defined as:

$$\hat{y}(u,v)=\mu +\alpha_{u}+\beta_{v}$$

Where *μ* is the model intercept, $\alpha_{u}$ is the main effect of UAS haplotype *u*, $\beta_{v}$ is the main effect of IHF2 haplotype *v*, and $y^{u,v}$ is the predicted Weighted Score.

Model parameters were estimated using training data only. Evaluation used a pair-held-out scheme in which all complete sequence records containing the same UAS–IHF2 haplotype pair were excluded together. UAS and IHF2 effects were then estimated from the remaining combinations. Only combinations for which both regional effects could be estimated from the training data were included.

An offset term was added where needed to represent the overall activity shift associated with the C_UAS_IHF2 background:

$$\hat{y}(u,v)=\mu +\gamma_{C}+\alpha_{u}+\beta_{v}$$

A low-rank interaction model was additionally evaluated as:

$$\hat{y}(u,v)=\mu +\alpha_{u}+\beta_{v}+p_{u}\cdot q_{v}$$

Where $p_{u}$ and $q_{v}$ are low-dimensional latent vectors for the UAS and IHF2 haplotypes, respectively. Interaction dimensionality, regularization strength, and other hyperparameters were determined using the training data or internal cross-validation.

Performance on pair-held-out combinations was evaluated using Spearman correlation, *R*^2^, RMSE, and MAE. Complete shared-haplotype and pair-held-out diagnostic results are shown in Supplementary Figure 4 and Supplementary Data 2.

### Mutual-information footprints and extreme-activity enrichment heatmaps

Mutual-information footprints and high-versus-low activity enrichment heatmaps used phenol-induced FACS–seq Weighted Scores as the activity phenotype and the complete 156-bp regulatory sequence as input.

Only A_UAS, B_IHF2, and C_UAS_IHF2, in which sequence positions retained a fixed coordinate correspondence, were included. F_random and D_IHF2_shift were excluded.

Activity classes were defined as low activity for Weighted Score <3.45, intermediate activity for 3.45 ≤ Weighted Score<3.65, and high activity for Weighted Score≥3.65. These classes were used only for mutual-information and enrichment analyses; regression models used the continuous Weighted Score.

Mutual information was calculated between nucleotide identity at each position and the three-level activity class. Position–nucleotide states supported by fewer than 10 sequences were marked as low support in the visualizations. Empirical *p* values from the position-level mutual-information analyses were adjusted for multiple testing using the Benjamini–Hochberg false-discovery-rate procedure within each predefined analysis set. For the extreme-activity enrichment analysis, multiple-testing correction was likewise applied across the tested position–nucleotide states within each analysis set. The manuscript-wide testing-family definitions are summarized in Supplementary Table 9.

For enrichment analysis, nucleotide frequencies were calculated separately in the high-and low-activity groups. Intermediate-activity sequences were excluded.

The extreme-bin enrichment for nucleotide b at position i was calculated as:

$$E_{i,b}=\log_{2}(\frac{\left(n_{\text{high},i,b}+1)(N_{\mathrm{low},i}+4\right)}{\left(n_{\mathrm{low},i,b}+1)(N_{\text{high},i}+4\right)})$$

Where $n_{\text{high},i,b}$ and $n_{\mathrm{low},i,b}$ are the counts of sequences carrying nucleotide b at position i in the high-and low-activity groups, respectively, and $N_{\text{high},i}$ and $N_{\mathrm{low},i}$ are the corresponding total supported sequence counts at that position.

### IHF2 repositioning and downstream-sequence analysis

IHF2 repositioning analysis was performed on D_IHF2_shift. The 13-bp consensus-matching IHF2 sequence at positions 123–135 of the 156-bp reference sequence, 5′-AAACAATACCTTG-3′, was used as the positional reference.

Variants in which the motif start differed from WT position 123 were assigned to D_IHF2_shift. Variants retaining the motif at positions 123–135 and containing most variation at positions 136–150 were assigned to E_IHF2_downstream.

Reconstruction direction, deletion length, compensatory-refill length, IHF2 displacement, and the number of additional nucleotide substitutions were extracted for each D_IHF2_shift variant. Displacement was defined as the difference between the motif start position in the variant and WT position 123.

Weighted Score distributions were compared across reconstruction categories using nonparametric tests. To determine whether reconstruction category explained activity beyond general sequence and sequencing-coverage differences, a baseline model included local sequence composition, the number of additional substitutions, and Input coverage. Variables representing IHF2 reconstruction direction and displacement were then added, and the increase in cross-validated *R*^2^ was calculated. The additional contribution of these reconstruction terms was assessed using a Freedman–Lane residual-permutation test. IHF2 site-shift and downstream-sequence analysis outputs are provided in Supplementary Data 3.

For E_IHF2_downstream, all local-sequence analyses were restricted to the final fixed-coordinate 15-bp sequence spanning positions 136–150. The primary position-specific additive model encoded each non-WT nucleotide state as a binary feature relative to the WT nucleotide at that position. These main-effect features were fitted using Ridge regression. The predicted activity for sequence x was defined as:

$${\mathrm{WS}}_{i}=\beta_{0}+\sum_{p=136}^{150}\sum_{b\neq {\mathrm{WT}}_{p}}\beta_{p,b}\cdot I(x_{i,p}=b)+\epsilon_{i}$$

A pairwise-interaction model extended the additive feature set with all supported pairwise position–nucleotide interaction terms and was fitted using strongly regularized ElasticNet regression. Its performance was compared with that of the additive Ridge model using the change in repeated cross-validated *R*^2^, and statistical significance was evaluated by permutation testing. Benjamini–Hochberg FDR correction was applied separately to the predefined families of downstream-sequence tests, including feature-level associations, position–nucleotide associations, and repeated-substitution direction tests. Prespecified global model comparisons and predictive-performance estimates were not treated as multiple-testing families. The correction families and reporting policy are summarized in Supplementary Table 9.

### Full-sequence regression models

The final prediction framework used only the complete 156-bp DNA sequence as input. Design-class labels, Input coverage, raw FACS-bin counts, experimental-batch labels, and single-clone measurements were not used as model inputs.

Batch 1 contained 11,940 non-WT variants with unique sequences and valid Weighted Scores. The data were jointly stratified by design class and within-class Weighted Score quintile and divided using random_state = 42 into training (*n* = 8,358), validation (*n* = 1,791), and held-out test (*n* = 1,791) sets.

The MLP combined position-specific one-hot sequence features with global 4-mer frequencies, whereas the Residual CNN operated directly on the 156 × 4 one-hot-encoded sequence. Both architectures were trained using the Batch 1 training set, with model selection based exclusively on validation-set performance. Full model configurations, random seeds, fixed splits, and inference outputs are provided in Supplementary Data 4.

N × 4 × 156 where *N* is the number of sequences, 4 is the nucleotide-channel dimension, and 156 is the regulatory-sequence length. The convolutional stem was followed by three residual blocks with channel configurations of 32 to 64, 64 to 128, and 128 to 128. The first two residual blocks were followed by max pooling, and the final block was followed by adaptive average pooling.

Both models used MSE loss, AdamW optimization, an initial learning rate of 3e-4, weight decay of 1e-4, a batch size of 256, a maximum of 80 epochs, and early stopping with a patience of 15 epochs. Each architecture was trained using seeds 42, 123, and 2026, and predictions were averaged across seeds.

Candidate models, including linear models, tree-based ensembles, boosting models, standard one-dimensional CNNs, Transformers, and BiLSTMs, were compared using only the training and validation sets. The MLP and Residual CNN both achieved high validation performance, and their equal-weight average achieved the best overall validation result. Fixed dataset splits, candidate-model predictions, and finalized ensemble outputs are provided in Supplementary Data 4.

To compare the full-sequence ensemble with simpler sequence representations, we additionally evaluated biologically structured regression baselines using the same fixed training, development, and held-out test framework. The additive baseline represented nucleotide identity using position-specific one-hot features. The structured pairwise model additionally included regularized nucleotide-state pair features within UAS, within IHF2, and across UAS–IHF2 combinations. A 4-mer Ridge baseline assessed predictive information attributable to short-sequence composition. Hyperparameters were selected using the fixed development set, and final performance was evaluated only on the held-out test set. Detailed definitions and performance metrics are provided in Supplementary Methods SM6 and Supplementary Table 8.

### Sequence-similarity and group-aware evaluation

To test whether model performance depended primarily on closely related sequences being present in the training set, we repeated model evaluation using sequence-grouped splits based on Hamming distance across the complete 156-bp sequence. Specifically, the ensemble was retrained under Hamming-1 connected-component and non-transitive center-radius-1 splits using the same training procedure as the primary model and was compared with nearest-neighbor regression baselines. Full split definitions, sample sizes, and performance metrics are provided in Supplementary Figure 7 and Supplementary Data 5.

### Batch 2 experimental processing and cross-batch evaluation

Batch 2 was performed 20 days after Batch 1 and was generated by an independent bacterial transformation of the same preconstructed plasmid-library pool used for Batch 1. Both formal FACS–seq experiments used 20 μM phenol for 8 h. Following transformation, Batch 2 was cultured, induced, FACS-sorted, recovered, sequenced, and processed separately from Batch 1. The same four-bin fluorescence-sorting framework and exact 156-bp whitelist-matching workflow were used for both batches. Detailed Batch 1 and Batch 2 sorting parameters, fluorescence-bin boundaries, sequencing statistics, and quality-control criteria are provided in Supplementary Table 6.

The formal Batch 2 analysis set contained 8,603 records, including one WT/control record. After exclusion of WT, 8,602 non-WT variants remained. Exact sequence matching identified 7,311 variants shared with Batch 1 and 1,291 variants unique to Batch 2. These counts define the primary Batch2_all, Batch2_shared, and Batch2_only model-evaluation subsets. A stricter bin-level QC analysis was performed separately for sensitivity testing. This analysis excluded 48 records with zero raw counts across Bin1-Bin4, yielding 8,555 analyzable Batch 2 records; within this strict-QC table, 7,278 shared non-WT variants and 1,277 Batch2-only variants were used for the corresponding Supplementary analyses. The strict-QC table was not mixed with the primary 8,602-variant model-evaluation denominator.

Overlapping sequences were used to evaluate agreement between experimental batches, whereas Batch 2-only sequences were used to evaluate predictions for complete sequences absent from Batch 1. Batch 2 measurements, subset definitions, cross-batch comparisons, and prediction outputs are provided in Supplementary Data 6.

### DeepSHAP attribution

DeepSHAP attribution was calculated separately for the MLP and Residual CNN using Batch 1 training-set reference backgrounds (Lundberg and Lee, 2017; Shrikumar et al., 2017). The interpretation manifest contained 1,000 sequences, comprising 500 Batch 1 held-out sequences and 500 Batch 2-only sequences. Reference backgrounds were selected only from the Batch 1 training set.

Three background sets, A, B, and C, each contained 96 training sequences sampled across six design classes and four within-class activity quartiles. SHAP values from the MLP and Residual CNN were calculated and interpreted separately and were not averaged into an ensemble SHAP value.

For the Residual CNN, position-level attribution was calculated from the SHAP value corresponding to the nucleotide observed in each sequence. For the MLP, the 624 position-specific one-hot features were summarized using the same procedure. The 256 4-mer features were analyzed separately because they did not have unique position assignments. DeepSHAP outputs and stability summaries are provided in Supplementary Data 8.

### In silico mutagenesis

Single-nucleotide ISM was performed using the locked equal-weight ensemble. Each non-parental substitution was generated explicitly and passed through the models for forward prediction.

A total of 1,000 reference sequences were scanned across all 156 positions and three alternative nucleotides, generating 468,000 mutation states. ISM effects were retained separately across sequence backgrounds. The predicted effect of substituting position *i* with nucleotide *b* was defined as:

ΔWS(i,b)=WS(mutant(i→b))−WS(reference)

Experimental effects were defined as E_Bin4 - E_Bin1, whereas ISM effects were defined as mutant prediction minus reference prediction. Formal comparisons included 264 UAS substitution states and 78 IHF2 substitution states. Spearman correlations, bootstrap confidence intervals, position-block permutation tests, and matched downsampling analyses were used to evaluate agreement. ISM and experiment-concordance outputs are provided in Supplementary Data 8.

### Single-clone validation

The validation panel contained 24 DmpR–Po promoter sequences, including six parent–variant pairs. Candidate sequences were commercially synthesized, cloned into the same reporter-plasmid backbone, transformed into BW25113, and confirmed by colony PCR and Sanger sequencing. Promoter sequences and summary measurements are listed in Supplementary Table 3, and complete validation data are provided in Supplementary Data 9.

Each clone was cultured in LB medium containing 100 μg/mL ampicillin. Induced cultures received 20 μM phenol, whereas uninduced cultures received an equal volume of solvent. Each independent culture well was treated as one biological replicate.

sfGFP fluorescence and OD_600_ were measured using a microplate reader. Technical readings were averaged within each biological well. Fluorescence was normalized by OD_600_, and candidate activity was further normalized to the corresponding plate-specific WT reference. The primary phenotype used for comparison with model predictions was log_2_-transformed relative induced activity. Uninduced relative activity was calculated from the corresponding solvent-treated biological wells using the same plate-specific normalization procedure. Induced/uninduced fold induction was calculated on the linear activity scale for each clone. These measurements were used only for clone-level basal-expression analysis and were not used to train or select the sequence-prediction models.

Condition-resolved summaries are provided in Supplementary Table 10; Supplementary Figure 17, and Supplementary Data 9.

## Data Availability

The raw amplicon sequencing data generated in this study have been deposited in the NCBI Sequence Read Archive (SRA) under BioProject accession PRJNA1529874 and SRA study accession SRP736915, with run accessions SRR40694477–SRR40694486. The sequencing data are available at https://www.ncbi.nlm.nih.gov/bioproject/PRJNA1529874. Code and reproducibility files supporting the analyses are available at https://github.com/xuyoci4-dev/DmpR_Po_reproducibility. Processed datasets supporting the analyses are provided in the Supplementary Material.
