# Supplementary material for: Sequence–activity mapping and model-guided engineering of the phenol-responsive DmpR–Po σ54 promoter
Source: Front Microbiol. 2026 Oct 7;17:1958192. doi: 10.3389/fmicb.2026.1958192 (PMC13682980; doi:10.3389/fmicb.2026.1958192)
Supplement: Supplementary file 1 [file Data_Sheet_1.docx]

# 1 Supplementary Material

DmpR–Po σ54 promoter sequence–activity mapping and machine-learning-guided validation

This Supplementary Material file follows the Frontiers supplementary-material template and contains Supplementary Figures, short Supplementary Tables, and a catalogue of machine-readable Supplementary Data packages.

# 2 Supplementary Methods

## 2.1 Batch 2 experimental provenance and processing

Batch 2 was performed 20 days after Batch 1. Both formal FACS-seq experiments used 20 µM phenol and an 8 h induction. Batch 2 used the same preconstructed plasmid-library pool as Batch 1, but an independent bacterial transformation and separate culture, induction, FACS, recovery, sequencing, and downstream processing. Batch 2 was not independently synthesized or independently cloned.

## 2.2  Batch 2 sequence matching and QC

Formal matching required an exact 156-bp whitelist match, A/C/G/T-only sequence, Input >= 5, total Bin1-Bin4 count > 0, pseudocount 1, channel-specific RPM normalization, and ordinal Weighted Score weights of 1, 2, 3, and 4. The formal table contained 8,603 records including WT/control, 8,602 non-WT sequences, 7,311 sequences shared with Batch 1, and 1,291 Batch2-only sequences. A separate strict Bin-total sensitivity membership set contained 8,555 records and 7,278 Batch1-Batch2 shared sequences; it was not substituted for the primary denominator.

## 2.3 Batch 2 coverage-bias analysis

Recovered_in_Batch2 was defined solely as exact membership in the formal 8,602-non-WT Batch 2 evaluation table. Batch 1 Weighted Score distributions were compared by ECDF, Cliff's delta, and Mann-Whitney testing. Design-class composition was assessed by contingency analysis and Cramer's V; a class-adjusted logistic model and an activity-by-design-class interaction assessed heterogeneity. Absence from the final Batch 2 table was not assigned to any particular synthesis, transformation, sorting, sequencing, or QC step.

## 2.4 Conditional fluorescence-weighting sensitivity analysis

Conditional fluorescence-weighting sensitivity used archived FITC-H population means for P3, P4, P5, and P6, assigned to sequencing Bin1, Bin2, Bin3, and Bin4, respectively, according to the confirmed gate-to-bin assignment. These weights were used only to test ranking sensitivity and were not interpreted as calibrated absolute fluorescence values.

## 2.5 Retention versus Hamming distance

Whole-sequence Hamming distance was calculated relative to the 156-bp WT sequence. Retention associations were modeled with design-class adjustment, with within-class analyses for D_IHF2_shift and F_random and designed-versus-retained positional mutation-frequency comparisons. These analyses do not infer synthesis-error causality.

## 2.6  Biologically structured regression baselines

Position-specific additive and structured pairwise regularized baselines, a 4-mer Ridge baseline, and the locked full-sequence ensemble were evaluated on the common 1,721-sequence held-out subset. Baseline hyperparameters were selected on the fixed development set without reference to held-out test data. Pairwise terms are statistical features and are not interpreted as molecular interactions.

## 2.7 Condition-resolved single-clone and parent-variant analyses

Condition-resolved GFP/OD600 values were summarized for WT and the 24 formally validated non-WT clones. Technical reads were first averaged within each biological well. Induced and uninduced activities were then separately divided by the corresponding same-plate WT reference; WT therefore equals 1 in each condition. Within-clone induction ratios were calculated as induced activity divided by uninduced activity. Six parent-variant comparisons used biological wells as the inferential unit. These clone measurements were not used for model training or model selection.

## 2.8  Multiple-testing strategy

Benjamini-Hochberg FDR correction was applied only within prespecified position/nucleotide hypothesis families when supported by the generating analysis. Global/regional tests and predictive metrics are reported with their defined resampling or model-evaluation procedures and were not pooled into a manuscript-wide FDR family.

# 3 Supplementary Results

## 3.1  Batch 2 coverage and recovery bias

Among 11,940 Batch 1 non-WT sequences, 7,311 were represented in the formal 8,602-non-WT Batch 2 set and 4,629 were not. Shared sequences had mean Batch 1 Weighted Score 2.4230 versus 2.2988 for Batch1-only sequences (mean difference 0.1242; Cliff's delta 0.0786, 95% CI 0.0580-0.0995). Recovery was strongly associated with design class (Cramer's V 0.4475) and the activity-by-design-class interaction was significant (LRT P = 8.933e-06); the overall activity shift was modest and should not be generalized as one common slope.

## 3.2 Conditional fluorescence-weighting sensitivity

Using the confirmed P3-P6 to Bin1-Bin4 assignment, the ordinal and conditional fluorescence-weighted rankings were highly concordant (Spearman rho = 0.987487). Top-10% and bottom-10% set overlaps were 92.7% and 86.4%, respectively. This supports rank robustness but does not calibrate physical fluorescence spacing.

## 3.3 Retention and Hamming distance

After design-class adjustment, retention showed a very small association with Hamming distance (OR 0.984 per additional mismatch, 95% CI 0.969-1.000; P = 0.049). Designed-versus-retained positional mutation frequencies remained correlated (Spearman rho 0.840; MAE 0.0455; RMSE 0.0475).

## 3.4 Structured sequence-baseline comparison

On the 1,721-sequence common held-out subset, the full-sequence ensemble outperformed additive, structured-pairwise, and 4-mer baselines while all models were evaluated on identical sequences.

## 3.5 Condition-resolved single-clone validation

Induced and uninduced GFP/OD600 summaries were available for WT and all 24 formally validated non-WT clones (Supplementary Figure S17 and Supplementary Table S10). Condition-resolved measurements demonstrate that clone outputs differed in both induced magnitude and induction ratio. Six parent-variant comparisons remain a limited, selected validation set and are not interpreted as a population-level engineering success rate.

# 4 Supplementary Figure legends

## 4.1 Supplementary Figure S1

Supplementary Figure S1. Preliminary phenol-response characterization, growth, and induction time course. (A) Time-resolved WT reporter fluorescence across phenol concentrations. (B) Response relative to the appropriate untreated control. (C) Growth/OD control measured alongside the reporter assay. This preliminary characterization is distinct from the formal FACS-seq induction condition, which used 20 µM phenol for 8 h in both Batch 1 and Batch 2.

## 4.2 Supplementary Figure S2

Supplementary Figure S2. NGS coverage, sequence filtering, and library retention. (A) Relationship between the 18,000 ordered oligopool entries, 17,500 unique designs, and final 11,940 retained non-WT Batch 1 sequences. The 17,500 unique designs comprised 17,499 non-WT sequences and one WT sequence; the additional 500 entries were repeated WT-control oligos. (B) Intended and retained non-WT sequence counts for the six official design classes. (C) Class-specific retention fractions; the dashed line indicates the overall non-WT retention fraction (11,940/17,499).

## 4.3 Supplementary Figure S3

Supplementary Figure S3. FACS gating, fluorescence binning, and Weighted Score calculation. Archived PBS, uninduced, and induced WT reference distributions are not presented as formal gate-defining controls. Formal fluorescence bins and score construction are assay-processing steps; exact historical gate boundaries are reported only where recovered from original records.

## 4.4 Supplementary Figure S4

**(A)**

**(B)**

Supplementary Figure S4. Cross-background UAS–IHF2 analyses and additive-model diagnostics. (A) Cross-background comparison of adjusted UAS and IHF2 effects. (B) Shared-haplotype transfer analysis. The panels quantify association across available sequence backgrounds and do not establish independent nucleotide-level causality.

## 4.5 Supplementary Figure S5

**(A)**

**(B)**

(C)

(D)

Supplementary Figure S5. Mutual-information, extreme-bin enrichment, and linkage controls. (A) UAS-A MI footprint and position-level uncertainty. (B) UAS-C MI footprint and position-level uncertainty. (C) UAS-A and UAS-C high-versus-low activity contrasts, including the alternative-base heat maps and complete four-bin enrichment summaries. (D) Criterion-6 linkage re-audit, including focal-alt/remote-WT co-occurrence, focal-alt/remote-alt co-occurrence, Cramer’s V/NMI summaries, effective-background distributions, and the revised sparse-background classification. These analyses describe associations in the designed library; they do not establish independent causal nucleotide effects.

## 4.6 Supplementary Figure S6

(B)

(C)

Supplementary Figure S6. Local diagnostics for composite IHF2-region reconstruction and downstream libraries. (A) Weighted Score distributions for D_IHF2_shift and E_IHF2_downstream variants. (B) Position-level downstream substitution effects with individual observations, effect estimates, sample sizes, and the zero-effect reference. (C) Existing Hamming-1 edge effects, position-level median effects, and connected-component size distribution for the E_IHF2_downstream sequence graph. The D designs jointly vary site displacement and reconstruction context; results are therefore interpreted as composite architecture-reconstruction effects.

## 4.7 Supplementary Figure S7

**(A)**

**(B)**

Supplementary Figure S7. Nearest-neighbor baselines and strict sequence-family holdout. (A) Nearest-neighbor, 1NN/kNN, and Hamming-distance analyses. (B) Connected-component and centre-radius-1 strict family holdouts. These evaluations test sensitivity to local sequence similarity and are distinct from random sequence-level splits.

## 4.8 **Supplementary Figure S8**

(A)

(B)

Supplementary Figure S8. Batch 2 repeatability and regional/class robustness analyses. (A) Batch 2 design-class-level Weighted Score distributions and median-rank comparison. (B) Sequence-level Batch 1 versus Batch 2 Weighted Score correspondence for the shared set. (C) Shared-haplotype transfer and context-versus-single-design-class comparisons. (D) Batch-centered regional effects for UAS and IHF2. These panels separate assay repeatability, haplotype transfer, and regional robustness; pure model-prediction scatterplots are presented in Supplementary Figure S9.

## 4.9 **Supplementary Figure S9**

(A)

(B)

(C)

(D)

Supplementary Figure S9. Batch 2 prediction and cross-batch consistency diagnostics. (A) Prediction-versus-observed score comparison in Batch 2. (B) Performance stratified by the stated Batch 2 subsets. (C–D) Cross-batch consistency diagnostics. Shared sequences quantify assay reproducibility, whereas Batch 2-only sequences quantify prediction under combined sequence and batch/condition shift.

## 4.10 Supplementary Figure S10

(E)

Supplementary Figure S10. Model comparison and design-class baseline analyses. (A) Design-class-only baseline. (B) Sequence-only model. (C) Sequence plus design class model. (D) Design-class-centered sequence model. (E) Batch 2-only extreme enrichment of the full-sequence ensemble ensemble, showing the locked 10% top and bottom subsets with 5,000-bootstrap 95% confidence intervals. The top-subset enrichment is 5.5001-fold and the bottom-subset enrichment is 7.0279-fold.

## 4.11 Supplementary Figure S11

Supplementary Figure S11. Weighted Score coverage and QC sensitivity. (A) Retained sequence count as the minimum Input-read threshold increases. (B) Design-class retention across the same thresholds. (C) Median absolute error stratified by Input-depth bin. The figure evaluates coverage-related label reliability and threshold sensitivity; it does not replace the prespecified primary QC threshold.

## 4.12 Supplementary Figure S12

**(A)**

**(B)**

**(C)**

**(D)**

(E)

(E)

Supplementary Figure S12. DeepSHAP and member-model attribution concordance. (A) MLP positional DeepSHAP attribution. (B) Residual CNN positional DeepSHAP attribution. (C) Cross-member positional concordance. (D) Agreement with member-level forward mutagenesis summaries. (E) Batch 1 held-out versus Batch 2-only regional attribution stability; the absolute positional-importance Spearman correlations are 0.978 for MLP and 0.989 for the Residual CNN (n = 156 positions). These are model-attribution stability analyses, not independent wet-lab validation. Because sequence variation was intentionally concentrated in UAS- and IHF2-associated regions by library design, attribution concentration in these regions is interpreted conditional on library design and not as independent discovery of regulatory architecture.

## 4.13 Supplementary Figure S13

**(A)**

(B)

(C)

(D)

Supplementary Figure S13. ISM–experiment concordance. (A) Region-stratified comparison between full-sequence ensemble forward in silico mutagenesis effects and FACS-derived substitution effects. (B) Position/base-level comparison for UAS (Spearman rho = 0.843, n = 264 tested substitution states) and IHF2 (Spearman rho = 0.675, n = 78). (C–D) Directional and background-dependence diagnostics. Because the model was trained on FACS-derived Weighted Scores, this concordance measures recovery of experimental statistical structure rather than fully independent biological validation.

## 4.14 Supplementary Figure S14

Supplementary Figure S14. Batch 2 coverage and recovery bias. (A) Exact 156-bp membership overlap: 7,311 Batch1-Batch2 shared sequences, 4,629 Batch1-only sequences, and 1,291 Batch2-only sequences. (B) Batch 1 Weighted Score ECDFs for shared and Batch1-only sequences. (C) Formal Batch 2 membership fraction by design class. (D) Within-class Cliff's delta estimates (shared minus Batch1-only). The overall activity difference is statistically detectable but modest; recovery is strongly design-class dependent and activity associations are heterogeneous by design class.

## 4.15 Supplementary Figure S15

Supplementary Figure S15. Conditional fluorescence-weighting sensitivity. (A) Ordinal Weighted Score versus conditional fluorescence-weighted score calculated from archived bin-specific RPM values. (B) Rank concordance. (C) Top- and bottom-decile overlap. P3, P4, P5, and P6 were assigned to sequencing Bin1-Bin4 according to the confirmed gate-to-bin assignment. The analysis tests robustness of sequence ranking and does not convert the conditional weights into calibrated absolute fluorescence.

## 4.16 Supplementary Figure S16

Supplementary Figure S16. Retention versus Hamming distance. (A) Intended and retained Hamming-distance distributions by design class. (B) D_IHF2_shift and F_random within-class Hamming associations. (C) Retention versus Hamming-distance relationship. (D) Designed versus retained positional mutation-frequency comparison. The adjusted association is very small and does not support a synthesis-error causal explanation for retention heterogeneity.

## 4.17 Supplementary Figure S17

Supplementary Figure S17. Same-plate WT-normalized fluorescence of WT and 24 validated promoter variants. (A) Induced and uninduced activities were separately normalized to the corresponding same-plate WT reference and are shown as log2 relative activity; the dashed line denotes WT = 0 (relative activity = 1). (B) Within-clone induced-to-uninduced activity ratio on a log2 scale. Technical reads were averaged within biological wells before condition summaries. These condition-resolved measurements were not used to train or select the sequence models.

# 5 Supplementary Tables

**Supplementary Table 2. Batch 1 sequencing, read processing, and quality-control summary**

| **Item** | **Value** | **Interpretation** |
| --- | --- | --- |
| Final retained variants | 11940 | After read processing and quality control |
| Sequence length | {156: 11940} | Retained sequences |
| Non-ACGT sequences | 0 | Excluded during sequence quality control |
| Input read threshold | Input >= 5 | Applied before final analysis |
| Zero total bin counts | 0 | No retained sequence with zero Bin1-Bin4 total |
| Weighted Score range | 1.005 to 3.927 | Final Batch 1 analysis set |
| Basecalling / image recognition | Illumina RTA software | Provider or pipeline metadata |
| Demultiplexing | Illumina bcl2fastq 2.20 | Provider or pipeline metadata |
| Raw data format | *.fastq.gz PF/raw data | Provider or pipeline metadata |
| Adapter trimming | Adapter sequences available in provider report; actual trimming/filtering performed by project scripts should be cited from Methods or code package. | Provider or pipeline metadata |

*Note. The complete read-level workflow and per-sequence QC data are provided as Supplementary Data.*

**Supplementary Table 1. Design rules and retained variant counts for the six DmpR–Po design classes**

| **Design class** | **Varied region** | **Design rule** | **Designed** | **Retained** | **Retention** |
| --- | --- | --- | --- | --- | --- |
| A_UAS | UAS (positions 10-53) | UAS sequence variation | 7000 | 6592 | 94.2% |
| B_IHF2 | IHF2-associated region (positions 123-135) | IHF2-associated sequence variation | 2500 | 1510 | 60.4% |
| C_UAS_IHF2 | UAS and IHF2-associated region | Joint UAS-IHF2 variation | 4000 | 2040 | 51.0% |
| D_IHF2_shift | IHF2 site reconstruction around positions 123-135 | IHF2 site-shift reconstruction | 994 | 487 | 49.0% |
| E_IHF2_downstream | IHF2-downstream region (positions 136-150) | Downstream sequence variation with IHF2 retained | 1006 | 622 | 61.8% |
| F_random | Distributed promoter sequence changes | Randomized promoter controls | 2000 | 689 | 34.4% |
| Total | - | - | 17500 | 11940 | 68.2% |

Note. Counts are sequence counts after the stated retention steps.

**Supplementary Table 3.** Promoter sequences and summary measurements for the 24-clone validation panel

| **Clone ID** | **Design class** | **Parent-variant** | **Role** | **MLP** | **ResCNN** | **Ensemble** | **Measured induced activity** | **n** | **Directional agreement** |
| --- | --- | --- | --- | --- | --- | --- | --- | --- | --- |
| DNA54_VAL_002 | A_UAS | 002 to 007 | Parent | 3.635 | 3.837 | 3.736 | 1.360 | 3 | Reference |
| DNA54_VAL_003 | C_UAS_IHF2 | 003 to 011 | Parent | 3.613 | 3.563 | 3.588 | 1.271 | 3 | Reference |
| DNA54_VAL_004 | E_IHF2_downstream | 004 to 013 | Parent | 3.659 | 3.500 | 3.579 | 1.164 | 3 | Reference |
| DNA54_VAL_005 | B_IHF2 | 005 to 030 | Parent | 3.529 | 3.421 | 3.475 | 0.974 | 3 | Reference |
| DNA54_VAL_006 | A_UAS | to | Single clone | 3.825 | 3.845 | 3.835 | 1.231 | 3 | Reference |
| DNA54_VAL_007 | A_UAS | 002 to 007 | Variant | 3.120 | 3.022 | 3.071 | -0.268 | 3 | Concordant |
| DNA54_VAL_008 | E_IHF2_downstream | to | Single clone | 3.488 | 3.478 | 3.483 | 0.724 | 3 | Reference |
| DNA54_VAL_010 | A_UAS | to | Single clone | 3.374 | 3.527 | 3.451 | 0.421 | 3 | Reference |
| DNA54_VAL_011 | C_UAS_IHF2 | 003 to 011 | Variant | 3.166 | 3.219 | 3.193 | 0.495 | 3 | Concordant |
| DNA54_VAL_013 | E_IHF2_downstream | 004 to 013 | Variant | 3.351 | 2.794 | 3.073 | 0.429 | 3 | Concordant |
| DNA54_VAL_014 | C_UAS_IHF2 | to | Single clone | 3.629 | 3.745 | 3.687 | 0.862 | 3 | Reference |
| DNA54_VAL_016 | E_IHF2_downstream | to | Single clone | 3.493 | 3.446 | 3.469 | 0.261 | 3 | Reference |
| DNA54_VAL_017 | A_UAS | 032 to 017 | Variant | 3.935 | 4.094 | 4.014 | 0.646 | 3 | Discordant |
| DNA54_VAL_018 | E_IHF2_downstream | to | Single clone | 3.325 | 3.470 | 3.397 | 0.391 | 3 | Reference |
| DNA54_VAL_019 | A_UAS | to | Single clone | 3.769 | 3.933 | 3.851 | 0.982 | 3 | Reference |
| DNA54_VAL_020 | C_UAS_IHF2 | 020 to 031 | Parent | 3.577 | 3.629 | 3.603 | 1.315 | 3 | Reference |
| DNA54_VAL_022 | E_IHF2_downstream | to | Single clone | 3.689 | 3.592 | 3.640 | 1.041 | 3 | Reference |
| DNA54_VAL_023 | A_UAS | to | Single clone | 0.994 | 0.953 | 0.974 | -3.564 | 3 | Reference |
| DNA54_VAL_024 | E_IHF2_downstream | to | Single clone | 1.202 | 0.971 | 1.087 | -2.544 | 3 | Reference |
| DNA54_VAL_025 | E_IHF2_downstream | to | Single clone | 1.266 | 1.018 | 1.142 | -2.537 | 3 | Reference |
| DNA54_VAL_026 | F_random | to | Single clone | 1.279 | 1.249 | 1.264 | -0.377 | 3 | Reference |
| DNA54_VAL_030 | B_IHF2 | 005 to 030 | Variant | 3.790 | 3.870 | 3.830 | 1.528 | 3 | Concordant |
| DNA54_VAL_031 | C_UAS_IHF2 | 020 to 031 | Variant | 3.914 | 4.075 | 3.995 | 1.400 | 3 | Concordant |
| DNA54_VAL_032 | A_UAS | 032 to 017 | Parent | 3.856 | 4.037 | 3.946 | 0.947 | 3 | Reference |

*Note. Predictions are Weighted Scores. Measured induced activity is log2(relative induced activity). n denotes biological replicates. Detailed well-level and technical-repeat data are supplied separately as Supplementary Data.*

**Supplementary Table 4.** Strains, plasmids, and reporter constructs used in this study

| **Name** | **Material type** | **Plasmid or insert** | **Use** |
| --- | --- | --- | --- |
| Escherichia coli BW25113 | Host strain | None | Cloning, promoter library screening, and single-clone validation host. |
| Escherichia coli BW25113 / pBR322-dmpR-Popr-GFP-WT | Single-clone WT reporter strain | pBR322-dmpR-Popr-GFP | Wild-type reporter control and within-plate normalization reference. |
| Escherichia coli BW25113 / po promoter oligopool | Promoter-variant library strain pool | po promoter oligopool | FACS-seq promoter library screening. |
| Escherichia coli BW25113 / pBR322-dmpR-Popr-GFP-DNA54_VAL_002 | Single-clone promoter-variant reporter strain | pBR322-dmpR-Popr-GFP | Individual clone validation of selected DmpR-Po promoter variant. |
| Escherichia coli BW25113 / pBR322-dmpR-Popr-GFP-DNA54_VAL_003 | Single-clone promoter-variant reporter strain | pBR322-dmpR-Popr-GFP | Individual clone validation of selected DmpR-Po promoter variant. |
| Escherichia coli BW25113 / pBR322-dmpR-Popr-GFP-DNA54_VAL_004 | Single-clone promoter-variant reporter strain | pBR322-dmpR-Popr-GFP | Individual clone validation of selected DmpR-Po promoter variant. |
| Escherichia coli BW25113 / pBR322-dmpR-Popr-GFP-DNA54_VAL_005 | Single-clone promoter-variant reporter strain | pBR322-dmpR-Popr-GFP | Individual clone validation of selected DmpR-Po promoter variant. |
| Escherichia coli BW25113 / pBR322-dmpR-Popr-GFP-DNA54_VAL_006 | Single-clone promoter-variant reporter strain | pBR322-dmpR-Popr-GFP | Individual clone validation of selected DmpR-Po promoter variant. |
| Escherichia coli BW25113 / pBR322-dmpR-Popr-GFP-DNA54_VAL_007 | Single-clone promoter-variant reporter strain | pBR322-dmpR-Popr-GFP | Individual clone validation of selected DmpR-Po promoter variant. |
| Escherichia coli BW25113 / pBR322-dmpR-Popr-GFP-DNA54_VAL_008 | Single-clone promoter-variant reporter strain | pBR322-dmpR-Popr-GFP | Individual clone validation of selected DmpR-Po promoter variant. |
| Escherichia coli BW25113 / pBR322-dmpR-Popr-GFP-DNA54_VAL_010 | Single-clone promoter-variant reporter strain | pBR322-dmpR-Popr-GFP | Individual clone validation of selected DmpR-Po promoter variant. |
| Escherichia coli BW25113 / pBR322-dmpR-Popr-GFP-DNA54_VAL_011 | Single-clone promoter-variant reporter strain | pBR322-dmpR-Popr-GFP | Individual clone validation of selected DmpR-Po promoter variant. |
| Escherichia coli BW25113 / pBR322-dmpR-Popr-GFP-DNA54_VAL_013 | Single-clone promoter-variant reporter strain | pBR322-dmpR-Popr-GFP | Individual clone validation of selected DmpR-Po promoter variant. |
| Escherichia coli BW25113 / pBR322-dmpR-Popr-GFP-DNA54_VAL_014 | Single-clone promoter-variant reporter strain | pBR322-dmpR-Popr-GFP | Individual clone validation of selected DmpR-Po promoter variant. |
| Escherichia coli BW25113 / pBR322-dmpR-Popr-GFP-DNA54_VAL_016 | Single-clone promoter-variant reporter strain | pBR322-dmpR-Popr-GFP | Individual clone validation of selected DmpR-Po promoter variant. |
| Escherichia coli BW25113 / pBR322-dmpR-Popr-GFP-DNA54_VAL_017 | Single-clone promoter-variant reporter strain | pBR322-dmpR-Popr-GFP | Individual clone validation of selected DmpR-Po promoter variant. |
| Escherichia coli BW25113 / pBR322-dmpR-Popr-GFP-DNA54_VAL_018 | Single-clone promoter-variant reporter strain | pBR322-dmpR-Popr-GFP | Individual clone validation of selected DmpR-Po promoter variant. |
| Escherichia coli BW25113 / pBR322-dmpR-Popr-GFP-DNA54_VAL_019 | Single-clone promoter-variant reporter strain | pBR322-dmpR-Popr-GFP | Individual clone validation of selected DmpR-Po promoter variant. |
| Escherichia coli BW25113 / pBR322-dmpR-Popr-GFP-DNA54_VAL_020 | Single-clone promoter-variant reporter strain | pBR322-dmpR-Popr-GFP | Individual clone validation of selected DmpR-Po promoter variant. |
| Escherichia coli BW25113 / pBR322-dmpR-Popr-GFP-DNA54_VAL_022 | Single-clone promoter-variant reporter strain | pBR322-dmpR-Popr-GFP | Individual clone validation of selected DmpR-Po promoter variant. |
| Escherichia coli BW25113 / pBR322-dmpR-Popr-GFP-DNA54_VAL_023 | Single-clone promoter-variant reporter strain | pBR322-dmpR-Popr-GFP | Individual clone validation of selected DmpR-Po promoter variant. |
| Escherichia coli BW25113 / pBR322-dmpR-Popr-GFP-DNA54_VAL_024 | Single-clone promoter-variant reporter strain | pBR322-dmpR-Popr-GFP | Individual clone validation of selected DmpR-Po promoter variant. |
| Escherichia coli BW25113 / pBR322-dmpR-Popr-GFP-DNA54_VAL_025 | Single-clone promoter-variant reporter strain | pBR322-dmpR-Popr-GFP | Individual clone validation of selected DmpR-Po promoter variant. |
| Escherichia coli BW25113 / pBR322-dmpR-Popr-GFP-DNA54_VAL_026 | Single-clone promoter-variant reporter strain | pBR322-dmpR-Popr-GFP | Individual clone validation of selected DmpR-Po promoter variant. |
| Escherichia coli BW25113 / pBR322-dmpR-Popr-GFP-DNA54_VAL_030 | Single-clone promoter-variant reporter strain | pBR322-dmpR-Popr-GFP | Individual clone validation of selected DmpR-Po promoter variant. |
| Escherichia coli BW25113 / pBR322-dmpR-Popr-GFP-DNA54_VAL_031 | Single-clone promoter-variant reporter strain | pBR322-dmpR-Popr-GFP | Individual clone validation of selected DmpR-Po promoter variant. |
| Escherichia coli BW25113 / pBR322-dmpR-Popr-GFP-DNA54_VAL_032 | Single-clone promoter-variant reporter strain | pBR322-dmpR-Popr-GFP | Individual clone validation of selected DmpR-Po promoter variant. |

*Note. All reporter strains used Escherichia coli BW25113. Antibiotic concentrations are reported where recorded in the source material.*

**Supplementary Table 5.** Primers, adapters, and indexing information used for library construction and amplicon sequencing

| **Name** | **Sequence (5' to 3')** | **Use** | **Stage** |
| --- | --- | --- | --- |
| prime-F | CGACGGCAGTGATTTTAGTATTAGA | Amplicon sequencing | PCR |
| prime-R | GCGCAGGACATCAAGCAA | Amplicon sequencing | PCR |
| P7 adapter (read1) | AGATCGGAAGAGCACACGTCTGAACTCCAGTCAC | Library adapter | Sequencing |
| P5 adapter (read2) | AGATCGGAAGAGCGTCGTGTAGGGAAAGAGTGT | Library adapter | Sequencing |

*Note. No sample-specific barcode or index was used. Adapter sequences are provider-reported standard adapter sequences rather than sample-specific barcodes.*

Supplementary Table 6. Batch 1 and Batch 2 experimental provenance, sequencing, QC, and overlap summary

| **Field** | **Batch 1** | **Batch 2** |
| --- | --- | --- |
| Upstream plasmid-library source | Preconstructed plasmid-library pool | Same preconstructed plasmid-library pool |
| Transformation | Formal Batch 1 transformation | Independent bacterial transformation |
| Relative experiment date | Reference | 20 days after Batch 1 |
| Host | Escherichia coli BW25113 | Escherichia coli BW25113 |
| Reporter plasmid | pBR322-dmpR-Popr-GFP | pBR322-dmpR-Popr-GFP |
| Phenol / induction | 20 µM / 8 h | 20 µM / 8 h |
| FACS strategy / bin boundaries | NOT RECOVERED | NOT RECOVERED |
| Sequencing reads / bases | NOT RECOVERED | NOT RECOVERED |
| Whitelist / QC | Exact 156-bp; Input >=5; total Bin >0 | Exact 156-bp; Input >=5; total Bin >0 |
| Weighted Score rule | Pseudocount 1; RPM; weights 1,2,3,4 | Pseudocount 1; RPM; weights 1,2,3,4 |
| Formal final dataset | 11,940 non-WT | 8,602 non-WT |
| Overlap | Reference | 7,311 shared; 1,291 Batch2-only |

Note. Values are limited to verified archived outputs; NOT RECOVERED denotes an unavailable original record rather than an inferred parameter.

Supplementary Table 7. Six parent-variant pair and clone robustness statistics

| **Pair** | **Exact edit** | **Predicted direction** | **Measured delta (log2)** | **Parent n** | **Variant n** | **Test** | **P** | **Direction** |
| --- | --- | --- | --- | --- | --- | --- | --- | --- |
| 002 to 007 | SHAP-disruptive edit | decrease | -1.620 | 3 | 3 | Welch | 0.00163 | yes |
| 003 to 011 | SHAP-disruptive edit | decrease | -0.775 | 3 | 3 | Welch | 0.0194 | yes |
| 004 to 013 | SHAP-disruptive edit | decrease | -0.728 | 3 | 3 | Welch | 0.0921 | yes |
| 005 to 030 | C21A+C27T | increase | 0.556 | 3 | 3 | Welch | 0.0399 | yes |
| 020 to 031 | C21A+C27T | increase | 0.079 | 3 | 3 | Welch | 0.799 | yes |
| 032 to 017 | G26A | increase | -0.322 | 3 | 3 | Welch | 0.225 | no |

Note. Values are limited to verified archived outputs; NOT RECOVERED denotes an unavailable original record rather than an inferred parameter.

Supplementary Table 8. Biologically structured baselines on the common held-out test subset

| **Model** | **Representation** | **Regularization** | **Test n** | **Spearman rho** | **R2** | **RMSE** | **MAE** | **Hyperparameter-selection set** |
| --- | --- | --- | --- | --- | --- | --- | --- | --- |
| Position-specific additive regularized ordinal baseline | WT-reference nucleotide-state indicators at all 156 positions; ordered four-bin response model | L2 regularized | 1721 | 0.853044 | 0.693014 | 0.386486 | 0.298727 | Fixed development set only |
| Structured pairwise regularized ordinal baseline | Additive nucleotide-state terms plus within-UAS, within-IHF2, and UAS x IHF2 supported nucleotide-state pairs; ordered four-bin response model | L2 regularized | 1721 | 0.898105 | 0.788482 | 0.320810 | 0.229793 | Fixed development set only |
| Position-aware 4-mer Ridge baseline | 468 WT-reference nucleotide-state indicators plus supported position-specific 4-mer indicators | Ridge (alpha = 1000) | 1721 | 0.902917 | 0.782163 | 0.325567 | 0.244359 | Fixed development set only |
| Locked equal-weight MLP-Residual CNN ensemble | Full 156-bp sequence; MLP 880-feature representation and Residual CNN 4 x 156 one-hot representation; equal 0.5/0.5 average | Locked ensemble | 1721 | 0.923253 | 0.845153 | 0.274490 | 0.197020 | Fixed development set before locked test |

Note. Values are limited to verified archived outputs; NOT RECOVERED denotes an unavailable original record rather than an inferred parameter.

Supplementary Table S9. Manuscript-wide multiple-testing policy and inferential families.

| **Analysis** | **Hypothesis family** | **Multiplicity control** | **Policy** |
| --- | --- | --- | --- |
| Module-wise Weighted Score global comparison | One prespecified omnibus comparison | No multiplicity correction | NO |
| Module-wise Weighted Score pairwise post-hoc comparisons | All 15 pairwise module comparisons in Fig2C_module_pairwise_statistics.csv | Benjamini-Hochberg FDR (column BH_FDR) | YES if individual pairwise significance is interpreted |
| UAS cross-background haplotype correlation | Single prespecified UAS regional test | No multiplicity correction | NO |
| IHF2 cross-background haplotype correlation | Single prespecified IHF2 regional test | No multiplicity correction | NO |
| Position-level mutual-information footprint | Within each Analysis_Set, separately across tested positions for base-identity MI and mutation-state MI | Benjamini-Hochberg FDR implemented by bh_fdr() | YES |
| Extreme-bin position–nucleotide enrichment | Within each Analysis_Set across all tested position × base contrast cells | Benjamini-Hochberg FDR implemented by bh_fdr(effect_p.ravel()) | YES |
| D_IHF2_shift covariate-adjusted architecture contribution | One prespecified global model comparison | No multiplicity correction | NO |
| E_IHF2_downstream feature associations | All 95 E-module feature associations in the formal table | Benjamini-Hochberg FDR (column fdr_bh_permutation) | YES |
| E_IHF2_downstream position–nucleotide association tests | All 60 position × base association tests in E_position_base_associations.csv | Benjamini-Hochberg FDR (column fdr_bh_permutation) | YES |
| E_IHF2_downstream Hamming-1 repeated substitution direction tests | All 51 observed repeated substitution-state sign tests | Benjamini-Hochberg FDR (column fdr_bh_sign_test) | YES |
| Downstream additive versus pairwise-interaction model comparison | One prespecified additive-versus-pairwise comparison | No multiplicity correction | NO |
| ISM versus experimental substitution-effect concordance: UAS and IHF2 | UNRESOLVED; likely the populated UAS/IHF2 concordance rows, but must be verified before wording as BH-FDR | FDR method is not named in the formal metric file or a recoverable source script: UNRESOLVED | YES if multiple dataset/subset concordance claims are presented as separate inferential findings |
| Position-block permutation analyses for ISM–experiment concordance | UNRESOLVED; report must name all executed region/subset tests | UNRESOLVED | NO for two prespecified regional tests; YES if all subsets/positions are inferentially claimed |
| Predictive performance metrics and bootstrap intervals | Not applicable unless a formal multi-model superiority test is made | No multiplicity correction | NO for estimation; YES only for a family of inferential model-comparison P values |

Supplementary Table S10. Condition-resolved fluorescence summaries for WT and 24 validated promoter variants.

| Clone_ID | Induced relative activity | Induced n | Uninduced relative activity | Uninduced n | Fold induction |
| --- | --- | --- | --- | --- | --- |
| WT | 1 | 3 | 1 | 3 | 1.31 |
| 002 | 2.57 | 3 | 0.346 | 3 | 7.41 |
| 003 | 2.41 | 3 | 0.352 | 3 | 6.86 |
| 004 | 2.24 | 3 | 0.341 | 3 | 6.58 |
| 005 | 1.96 | 3 | 0.357 | 3 | 5.51 |
| 006 | 2.35 | 3 | 0.226 | 3 | 10.4 |
| 007 | 0.83 | 3 | 0.325 | 3 | 2.55 |
| 008 | 1.65 | 3 | 0.221 | 3 | 7.47 |
| 010 | 1.34 | 3 | 0.23 | 3 | 5.81 |
| 011 | 1.41 | 3 | 0.227 | 3 | 6.21 |
| 013 | 1.35 | 3 | 0.219 | 3 | 6.14 |
| 014 | 1.82 | 3 | 0.215 | 3 | 8.45 |
| 016 | 1.2 | 3 | 0.216 | 3 | 5.56 |
| 017 | 1.57 | 3 | 0.221 | 3 | 7.08 |
| 018 | 1.31 | 3 | 0.212 | 3 | 6.2 |
| 019 | 1.97 | 3 | 0.416 | 3 | 4.75 |
| 020 | 2.49 | 3 | 0.33 | 3 | 7.55 |
| 022 | 1.96 | 3 | 0.0837 | 3 | 23.4 |
| 023 | 0.0845 | 3 | 0.0624 | 3 | 1.36 |
| 024 | 0.171 | 3 | 0.123 | 3 | 1.4 |
| 025 | 0.172 | 3 | 0.131 | 3 | 1.31 |
| 026 | 0.77 | 3 | 0.331 | 3 | 2.33 |
| 030 | 2.88 | 3 | 0.359 | 3 | 8.03 |
| 031 | 2.64 | 3 | 0.35 | 3 | 7.54 |
| 032 | 1.93 | 3 | 0.343 | 3 | 5.63 |

# 6 Supplementary Data packages

## 6.1 Formal Supplementary Data package files

| **No.** | **Title** | **Format** | **Content** |
| --- | --- | --- | --- |
| 01 | Library design and Batch 1 sequence–activity data | ZIP | Supplementary_Data_01_library_and_Batch1.zip; whitelist, sequences, Batch 1 counts, Weighted Scores, and QC fields. |
| 02 | UAS–IHF2 haplotype, additive-model, and mutual-information outputs | ZIP | Supplementary_Data_02_haplotype_additive_MI.zip; haplotype transfer, additive models, MI, enrichment, and linkage controls. |
| 03 | IHF2 site-shift and downstream-sequence analysis outputs | ZIP | Supplementary_Data_03_IHF2_architecture_and_downstream.zip; reconstruction annotations, downstream effects, local models, and sequence-neighbor analyses. |
| 04 | Dataset splits, model predictions, and finalized full-sequence models | ZIP | Supplementary_Data_04_splits_predictions_models.zip; fixed splits, predictions, comparisons, finalized models, and reproducibility code. |
| 05 | Nearest-neighbor and strict sequence-family holdout outputs | ZIP | Supplementary_Data_05_neighbor_and_strict_holdout.zip; group-aware splits, baselines, predictions, and distance-stratified metrics. |
| 06 | Batch 2 measurements, cross-batch comparisons, and prediction outputs | ZIP | Supplementary_Data_06_Batch2_measurements_and_predictions.zip; Batch 2 subsets, shared-sequence comparisons, predictions, and enrichment. |
| 07 | Weighted Score reliability and QC-sensitivity outputs | ZIP | Supplementary_Data_07_Weighted_Score_QC_sensitivity.zip; depth, pseudocount, uncertainty, and threshold-sensitivity results. |
| 08 | DeepSHAP, in silico mutagenesis, and experiment-concordance outputs | ZIP | Supplementary_Data_08_DeepSHAP_ISM_concordance.zip; neural attribution, ISM, stability, and experimental concordance. |
| 09 | Single-clone validation data | ZIP | Supplementary_Data_09_clone_validation.zip; 24-clone sequences, predictions, measurements, normalization, and pair directions. |
| 10 | Source manifests and auxiliary curated outputs | ZIP | Supplementary_Data_10_source_manifests_and_auxiliary_outputs.zip; package manifest, checksums, mapping, and reproducibility notes. |
